# New Approaches and Strategies for the Repurposing of Iron Chelating/Antioxidant Drugs for Diseases of Free Radical Pathology in Medicine

**DOI:** 10.3390/antiox14080982

**Published:** 2025-08-10

**Authors:** George J. Kontoghiorghes

**Affiliations:** Postgraduate Research Institute of Science, Technology, Environment and Medicine, Limassol 3021, Cyprus; kontoghiorghes.g.j@pri.ac.cy; Tel.: +357-26272076

**Keywords:** iron chelating/antioxidants drugs, drug repurposing, deferiprone, deferoxamine, deferasirox, N-acetylcysteine, free radical pathology, ferroptosis, clinical trials

## Abstract

There is an urgent need for new approaches and strategies for the introduction of antioxidant drugs in medicine. Despite hundreds of clinical trials with potential antioxidants, no antioxidant drugs have so far been developed for clinical use; this is mainly as a result of commercial reasons, but also due to insufficient data for regulatory authority approval. Antioxidant activity is a physiological process essential for healthy living. However, increased production of toxic free radicals and reactive oxygen species is observed in many clinical conditions, which are associated with serious and sometimes irreversible damage. Antioxidant drug strategies may involve short- to long-term therapeutic applications for the purpose of prevention, treatment, or post-treatment effects of a disease. These strategies are different for each disease and may include the design of protocols for the inhibition of oxidative damage through iron chelation, enhancing antioxidant defences by increasing the production of endogenous antioxidants, and activating antioxidant mechanisms, as well as the administration of synthetic and natural antioxidants. Both the improvement of antioxidant biomarkers and clinical improvement or disease remission are required to suggest effective therapeutic intervention. More concerted efforts, including new academic strategies, are required for the development of antioxidant drugs in clinical practice. Such efforts should be similar to the fulfilment of orphan or emergency drug regulatory requirements, which, in most cases, involve the treatment or clinical improvement of rare or severe diseases such as neurodegenerative diseases and cancer. Promising results of antioxidant therapeutic interventions include mainly the repurposing of the iron chelating/antioxidants drugs deferiprone (L1) and deferoxamine, and also the iron-binding drug N-acetylcysteine (NAC). In some clinical trials, the lack of pharmacodynamic and ferrikinetic data, wrong posology, and insufficient monitoring have resulted in inconclusive findings. Future strategies involving appropriate protocols and drug combinations, such as L1 and NAC, appear to improve the prospect of developing antioxidant drug therapies in different diseases, including those associated with ferroptosis. New strategies may also involve the use of pro-drugs such as aspirin, which is partly biotransformed into iron chelating/antioxidant metabolites with chemopreventive properties in cancer, and also in other therapeutic interventions. A consortium of expert academics on regulatory drug affairs and clinical trials could increase the prospects for antioxidant drug development in medicine.

## 1. Introduction

Oxygen is an essential element for humans and other aerobic organisms, as it is mainly required for respiration and also many other functions. Oxygen is also a reactive element, causing oxidative and other related processes in living organisms [1,2]. In some of these processes, reactive free radicals (FR), which are chemical compounds with unpaired electrons, and other reactive oxygen species (ROS) are naturally formed and participate in physiological reactions and pathways. Naturally occurring FRs include superoxide, nitric oxide, and hydroxyl radical, whereas ROSs include hydrogen peroxide and other peroxides such as lipid peroxides [1,2,3,4]. Under normal physiological conditions, some of these metabolic pathways involving FRs and ROSs participate in the metabolism of natural compounds, xenobiotic molecules (including drugs), the oxidation of food products, the regulation of the circadian clock, cell signalling, ageing, and ferroptosis [3,4,5,6,7,8].

Under normal physiological conditions, the regulation of different reactions involving FRs and ROSs is strictly controlled by pathways, which involve antioxidant mechanisms for the maintenance of redox homeostasis and also repair systems for the restoration of related damages [1,2,3,4,5,6,7,8]. The antioxidant mechanisms also involve enzymes such as superoxide dismutase, glutathione peroxidase, and catalase, and also natural antioxidant molecules such as glutathione and dietary molecules including vitamins A, C, and E, and also various polyphenols [1,2,3,4,9,10,11].

Free radicals and ROSs have variable reactivity and half-lives. Some of them can react with almost all biomolecules, including DNA, sugars, lipids, and proteins, and can cross cell membranes and intracellular compartments where they can react rapidly, especially with biomolecules present in their vicinity [1,10]. It is estimated, for example, that under normal physiological conditions, about 90% of FRs in cells are produced in the mitochondria during energy transduction, without causing toxic side effects to the other organelles or within cells [12,13]. However, functional and structural abnormalities or damage to mitochondria and other organelles can cause an increase in the production of toxic quantities of FRs, which can result in serious and sometimes irreversible damage to the cells and the tissues affected. For example, mitochondrial malfunction has been identified in many genetic and acquired diseases, cancer, and ageing [12,13,14].

Redox homeostatic imbalance could be caused by many other factors in addition to mitochondrial damage, including, for example, endogenous factors such as the presence of excess iron or copper in different body organs, and also by exogenous factors such as exposure to radioactivity, heat, chemical reactants, heavy metals, X-rays, and UV irradiation [15,16,17,18,19,20,21,22]. In such cases, an increase in FR and ROS production, and also of associated cascades of related molecules is observed, which under normal conditions, can be reversed by an innate effective antioxidant system. In this context, the main function of the antioxidant system, which is composed mainly of antioxidant enzymes, molecules, and mechanisms, is to maintain redox balance by preventing, delaying, or neutralising the harmful effects of FRs and ROSs [1,2,9,10,11,17,18].

The production of excess FRs and ROSs can also cause oxidative stress in cells, which can lead to oxidative stress toxicity (OST), unless an effective antioxidant control in the affected cells is present and redox balance is restored [1,2,3,4,10]. However, prolonged exposure to excess FRs and ROSs could lead to irreversible toxicity and serious damage. In particular, OST is capable of causing mainly oxidation-related damaging modifications to biomolecules, including DNA, sugars, lipids, and proteins, and may also progressively result in structural and functional damage to organelles, cells, tissues, and organs [1,2,3,4,10,23,24,25,26,27].

Typical examples of redox imbalance and irreversible FR and ROS damage are during rancidification, cell necrosis, and the period after death [2,3,27]. In the latter case, and during the decomposition of the body, the antioxidant system and repair mechanisms progressively become obsolete, resulting in a rapid increase in the rate of FR and ROS production and related cascades, which could reach uncontrollable levels, leading to accelerated and irreversible biomolecular, cellular, and tissue damage. Similar observations of accelerated oxidative damage have been identified in many diseases and pathological conditions, where there is a variable degree of OST-related tissue damage due to the overproduction of FRs and ROSs, while at the same time, there is also the inability of the antioxidant system to contain or reverse the damage [2,3,4,10,27].

The maintenance of redox homeostasis and the antioxidant protection mechanisms appear to be essential for the survival of humans and other living organisms. Similarly, the regulatory control of the biomolecules, metal ions, enzymes, metabolic pathways, redoxomic, and other factors involved in redox homeostasis and antioxidant protection are also essential for normal physiological function. In this context, specific scavenging functions are performed for the neutralisation of excess FRs and ROSs in cells. For example, during the production of excess toxic levels of hydrogen peroxide in cells, an increased utilisation of intracellular reduced glutathione (GSH) is involved, which, in a reaction with hydrogen peroxide, forms water and an oxidised glutathione dimer (GSSG). This reaction, and more specifically the ratio of GSH to GSSG, is often used as a measure of the level of oxidative stress in cells or other compartments [23,28]. Similarly, vitamins A, C, and E; polyphenols; and other dietary antioxidants react with FRs and form less reactive FR intermediates, which subsequently can cause fewer or no damaging effects to other biomolecules [10,23,28,29,30,31].

A major component required for the maintenance of redox homeostasis in cells is the presence and control of the metabolism of the essential transition metals iron and copper. Both of these metal ions play an important catalytic role for the production of FRs in biological systems, whether in metalloproteins in mitochondria or as metalloenzymes, or in the form of low molecular weight metal complexes [10,23,32,33]. The metabolism of both of these metal ions is strictly regulated not only because they are essential components contributing to the maintenance of redox homeostasis, but also because of their participation in many other physiological functions and activities [32,33,34]. In particular, the essential role of iron and cell membrane lipid peroxidation in ferroptosis, a programmed cell death process recently identified in all types and stages of cancer, as well as all other diseases, highlights the critical role of iron metabolism in normal and disease states [35,36,37,38,39,40]. Similar mechanisms apply in the role of copper in cuproptosis and implications of related diseases [41,42,43].

The absorption, transport, storage, and utilisation of iron and copper are controlled by different metabolic pathways involving specific proteins. For example, transferrin can mobilise and carry up to two molecules of iron in blood and is regarded as a powerful natural chelator/antioxidant with antimicrobial and other properties [33,34,44]. Furthermore, the iron storage protein ferritin is found in all the cells and can store up to 4500 molecules of iron in the form of polynuclear oxohydroxy–iron complexes, which are not normally involved in redox reactions [33,34,45]. However, under certain conditions (e.g., in cellular damage or in ferroptosis), labile iron is released, which can catalyse FR reactions and cascades, leading to a vicious circle of OST and damage [33,34,45]. In contrast, antioxidants and other molecules, including natural and synthetic chelators that could bind iron strongly, may potentially inhibit the iron-catalysed FRs and ROSs and potentially inhibit OST and damage, as well as ferroptosis [10,33,34,35].

Metal-binding ligands and chelators play many important roles not only in biology, but also in medicine [46]. In biological systems, metal ions, including iron and copper, are always found bound to ligands containing the electron donor atoms oxygen, nitrogen, and sulphur. These three atoms are involved in coordinating covalent bond formation with the metal ions. In this context, all iron and copper, or other metal-associated biological processes and activities, are expressed and function through metal binding with different ligands [33,46].

Chelators (Chele, Greek χειλή-claw of a crab) are organic molecules possessing two or more ligands which have high affinity and can bind metal ions, forming a chelator–metal complex composed of a ring with the metal ion as the closing member [10,33,44]. The affinity of various ligands and the stability of the iron or other metal ion–chelator complexes are different, with specific physicochemical, pharmacological, functional, and other characteristics in each case. These differences could lead to a continuous competition between metal ions for ligand and chelating binding sites in biological systems [10,33,46,47]. In particular, effective iron chelators could bind and inhibit redox-active iron participating in the Fenton reaction and other iron catalytic centres, associated with FR pathology and tissue damage in many different clinical conditions, as well as diseases associated with ferroptosis [10,33,46].

Overall, chelators and chelator–metal complexes play very important redox, biological, and clinical roles in normal and pathological conditions. Furthermore, the development of iron chelating/antioxidant drugs could have a major impact on the treatment of diseases associated with FR pathology, and also play a pivotal role in the regulation of ferroptosis and all associated diseases. The main purpose of this review is to suggest new approaches and strategies for the repurposing of iron chelating drugs and also other drugs with antioxidant properties for the treatment of different diseases associated with free radical pathology.

## 2. Developmental Aspects of Potential Antioxidant Drugs for Clinical Use

The development of antioxidant drugs is a major challenge for many investigators involved in many fields of medical sciences, including all clinical conditions, pharmacology, toxicology, and nutrition. Millions of publications have described the damaging effects of FR/ROSs in different aspects of almost all clinical conditions, including, for example, DNA damage and as a cause of cancer, neuronal damage and neurodegenerative diseases, as well as ageing. Similarly, the involvement of redox changes has been widely discussed in relation to the mode of action of many drugs, nutraceuticals, natural compounds, and food components [48,49]. However, despite the availability of such large amounts of information on FR/ROSs in medicine, and antioxidant activity by many drugs and natural compounds, there has not been a serious systematic effort for the development of antioxidant drugs for any diseases through the drug regulatory route. This procedure is necessary for the introduction of all drugs intended for clinical use, with specific regulatory requirements in each case, in particular by the EMA in Europe and the FDA in the USA [50].

### 2.1. Limitations in the Use of Repurposed Drugs and Nutraceuticals in Medicine

There are many paradoxes related to the use of antioxidants in medicine, especially in conditions related to FR pathology. One of the paradoxes is that no pharmaceutical drugs are yet available or prescribed for antioxidant therapies in clinical practice for any clinical condition. This paradox is observed despite the fact that hundreds of thousands of publications in many sectors of science, including chemistry, biochemistry, pharmacology, toxicology, and medicine, implicate FR/ROSs and OST damage in almost all pathological conditions, including cancer, neurodegeneration, cardiac, liver, kidney disease, etc.

Another major paradox is the wide use of antioxidant nutraceuticals in traditional folk medicine, which has been evolved into a multibillion-euro industry worldwide [49]. However, despite the commercial use of many natural antioxidants such as nutraceuticals, mainly in the form of dietary supplements such as vitamin C, quercetin, curcumin, etc., the overall results of a number of clinical trials suggest that as of yet, there is insufficient clinical evidence, including robust clinical findings that such antioxidants can be introduced in clinical practice. In particular, no satisfactory evidence has been submitted to the regulatory drug authorities that such antioxidants can offer effective treatments or improvements in any clinical condition. In this context, more efforts, including the fulfilment of regulatory drug requirements, are generally needed for the introduction of antioxidant drugs in medicine [49,50].

In almost all cases of pharmaceuticals, the design and development of any single drug is a tedious, time-consuming, and very expensive process, which is mainly undertaken by pharmaceutical companies based mostly on commercial criteria [50]. On scientific grounds, the regulatory approval of a new drug is based on diagnostic, therapeutic, safety, and other criteria. In such cases, and as it has been previously shown with other drugs, sufficient information should be provided to regulatory drug authorities regarding the efficacy, low toxicity, and advantages of the proposed candidate antioxidant drug over other approved drugs (if any) used for the treatment of a specific disease. Such information includes sufficient data related to the proposed drug from chemical synthesis and chemical properties to pre-clinical studies, toxicology, and clinical trials [50].

Despite the overwhelming scientific information and public interest in the health benefits of the use of antioxidants, the pharmaceutical industry appears, in general, reluctant to be involved in the development of antioxidant pharmaceuticals. In the absence of interest in antioxidant drug development by the pharmaceutical companies, a number of new strategies could be considered for drug development, which, for example, could be based on existing legislation regarding orphan drugs, emergency drugs, or generic drug repurposing. These latter three drug developmental processes are generally more flexible and less expensive in comparison to the development of a new synthetic drug. In particular, the major efforts for the development of pharmaceutical antioxidants could be facilitated by targeting, for example, FR/ROSs and OST damage in diseases where there are no available effective treatments, such as cancer, ischemia-reperfusion injury, renal, neurodegenerative, and other similar conditions. This process can further be facilitated by the identification of a specific antioxidant target(s) related to FR/ROSs and OST, which may involve a particular toxicity aspect in a procedure or in organ or in specific cells, sub-cellular compartment, metabolic pathway, etc. In each such targeted case, a significant improvement or remission should be shown in a disease in comparison to a placebo using the stepwise phases (I-IV) of drug development, including double blind randomised clinical trials [50].

The targeting of FR toxicity in each clinical condition is a complicated process which may involve many stages from the initiation stage to the FR cascade stage, and also one or more body compartments. Similarly, each proposed antioxidant drug has specific pharmacological properties and a different mode of action, as well as target organ(s). In this context, it is unlikely that a single potential antioxidant drug will be effective at all the stages of FR toxicity, and also for all affected body compartments. However, several aspects of antioxidant targeting may have a better outcome than other approaches for decreasing FR toxicity. Such targeting strategies may involve the prevention of excess FR production, inhibition of the initiation stage of FR production, and the use of different drugs affecting different stages of FR toxicity.

Another antioxidant targeting strategy may involve antioxidant combination therapy. In many cases, drug combination therapies are aimed at increasing the overall efficacy and/or decreasing the overall toxicity in comparison to a single drug therapy (monotherapy) [33,46]. In almost all the cases of drug development for the treatment of each disease, pharmaceutical companies promote the use of their drug while at the same time discourage its use in combination therapies of similar mode of activity drugs by other pharmaceutical manufacturers, claiming possible toxicity interactions. Within this context and in practice, drug combination therapies, in most cases, including iron chelation therapy arise mostly from academic initiatives. Since all drugs differ in physicochemical, pharmacological, toxicological, and other properties, their mode of action affects each target to a variable extent. Similar effects are expected in the use of antioxidants for different contributory metabolic pathways, cellular compartments, and organs, which may be involved in the FR pathology in each disease.

Several other factors could affect the efficacy and toxicity of an antioxidant drug in each clinical condition, including different forms of interactions. For example, the interaction of an antioxidant drug with other antioxidant drugs in combination therapies and also other drugs used for the treatment of the underlying disease, as well as interactions with dietary molecules such as ascorbic acid, may all affect the mode of action of a proposed therapeutic antioxidant and also the overall outcome of the antioxidant therapy [47,51].

In general, specific targeted antioxidant therapies could be developed based on the characterisation of the causes, mechanisms, and pathways of FR/ROS and OST damage, and also the selection of the appropriate drugs and drug combinations that could have access to the site of the toxicity or damage. Overall, the selected antioxidant drug or drug combinations should be shown to prevent or reverse oxidative toxicity-related damage, while at the same time, a concomitant clinical improvement or remission of the associated pathological condition is observed in the affected patients.

### 2.2. General Characteristics and Requirements for Antioxidant Drugs in Medicine

There have been many efforts over the last decades for the development of antioxidant drugs. In most cases, the emphasis has mainly been focused on the selection of repurposed generic drugs, which have already been used in the treatment of other diseases and also fulfilled many of the drug regulatory requirements for clinical use. Furthermore, nutraceuticals are another promising class of natural compounds with potential for clinical development as antioxidant drugs, where, in some cases, relaxed drug regulatory requirements may also apply because of satisfactory safety levels following previous long-term use in humans.

There are many drug regulatory controversies and limitations in relation to the use of nutraceuticals or repurposed pharmaceuticals as antioxidant drugs in medicine. In particular, a lot of information regarding the drug regulatory requirements for use in each disease, such as risk/benefit assessment, therapeutic index, pharmacology, and toxicology, is still lacking or is insufficient [50,52]. Furthermore, clinical evidence for a specific FR/ROS targeting and improvement or treatment of a specific clinical condition, including posology parameters, is also, in most cases, unavailable or not conclusive for optimal use in different categories of patients [52].

Despite the many limitations, substantial information on safety, efficacy, and other pre-clinical and clinical parameters, including clinical trials, post-marketing surveillance, and long-term clinical use results is generally more readily available regarding repurposed generic drugs, which are intended for clinical use as antioxidant drugs [50,52]. This information is available because of the fulfilment of extensive tasks regarding regulatory drug approval requirements and also, in many cases, long-term post-marketing monitoring of adverse effects of generic drugs in other disease(s).

There are hundreds of examples of pharmaceuticals, which are known to be involved in redox reactions in the medical literature [51,52]. Many of these are also known to have antioxidant properties and some clinical potential for possible antioxidant applications in medicine. In each pharmaceutical drug case, the characterisation of the potential clinical antioxidant drug activity could be identified by several parameters, including the mechanism of antioxidant action, the effects on specific target(s), pharmacological effects, toxicological effects, and, most importantly, diagnostic evidence of the improvement or treatment in each clinical condition [23,28,33,52].

The consideration of several new parameters may be envisaged during the repurposed antioxidant drug application in each disease, such as antioxidant drug application and posology for prophylaxis, long- or short-term therapy, topical or systemic use, timing of administration, pharmacological and toxicological limitations, etc. Furthermore, specific considerations may also apply in the administration of antioxidant drugs as adjuvant therapy in many conditions, for example, following surgery, conditions of hypoxia, tissue damage, drug toxicity, etc.

Drug interactions are another parameter which may influence the selection of effective antioxidant drugs for different diseases. In such cases, there could be a range of effects, including positive and negative therapeutic implications [51]. For example, the selection of antioxidant drug combinations could be more effective than single antioxidant drug therapy. In contrast, there could be increased toxicity or reduced efficacy in cases of the interaction of the antioxidant drug with other therapeutic drugs used in the same clinical condition [50,51].

Individual variations in the level of OST and the response to drug antioxidant treatment, other considerations and factors in the context of personalised medicine, increase further the complexity and assessment of antioxidant drug development. Such factors include the malfunction or insufficiency of the innate antioxidant pathways and mechanisms, dietary habits, other diseases, infection, the state of the immune system, organ function, age, etc.

Overall, there are many requirements, parameters, and limitations in the selection of potential antioxidant drugs for the specific targeting of each pathological condition related to FR/ROS toxicity. Similarly, there are many possibilities for the interaction of the potential antioxidant drug with other drugs or natural compounds. One of the major classes of potential repurposed antioxidant drugs is the iron chelating drugs, which have increased prospects for development and clinical use in many diseases.

## 3. Iron Chelating Drugs with Antioxidant Effects in Medicine

Hundreds of drugs have metal-binding ligands and chelating sites in their chemical structures, each with different iron-binding potential and also involvement in redox interactions. Some of these interactions include iron chelating/antioxidant activity and the particular inhibition of the Fenton reaction, where iron acts as a catalyst for the production of FRs [10,23,32,51,52]. In general, there is wide variation in the iron-binding affinity and the redox effects of such drugs, as well as wide differences in their pharmacological, toxicological, and other properties. Furthermore, other factors such as interactions with other drugs, drug metabolites, dietary molecules, and other metal ions may also affect their iron-binding and redox effect potential [33,46,47].

**Figure 1:**
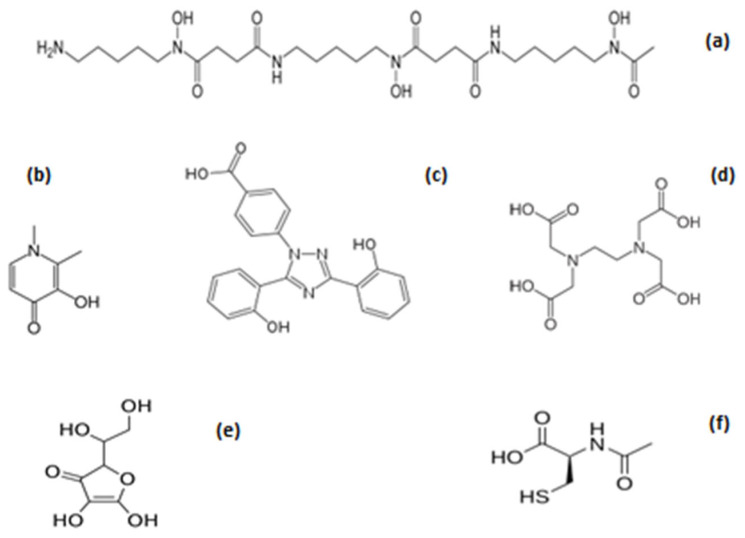
The chemical structure of the iron chelating drugs and other drugs with iron-binding capacity. The iron chelating drugs deferoxamine (**a**), deferiprone (**b**), and deferasirox (**c**) are widely used for the treatment of iron overload and have a potent antioxidant capacity. EDTA (**d**) is widely used for heavy metal detoxification and in alternative medicine, and also has iron-binding capacity. Vitamin C or ascorbjc acid (**e**) is a natural product and nutraceutical widely used as an antioxidant and has iron-binding capacity. N-acetylcysteine (**f**) is a drug used as an antidote for paracetamol toxicity overdose, with antioxidant properties and has iron-binding capacity.

The molecular features and properties of the iron chelating drugs deferiprone (L1), deferoxamine (DF), and deferasirox (DFRA) are of great interest for designing repurposing strategies for antioxidant activity, especially in relation to their iron-binding/redox effects (Figure 1). Similarly, several other widely used drugs and nutraceuticals and/or their metabolites, all of which possess iron-binding ligands and chelating sites involved in redox effects, also attract major interest for repurposing and possible application as antioxidant drugs for use in diseases associated with FR pathology and also for diseases associated with ferroptosis.

### 3.1. The General Iron Chelating Properties of Deferiprone, Deferoxamine and Deferasirox

The iron chelating drugs L1, DF, and DFRA are primarily and widely used for the mobilisation and excretion of excess iron from the body, which is a life-saving treatment for transfusional iron overload in thalassaemia major (TM) and other similar iron-loaded clinical conditions (Figure 1) [53,54,55,56,57,58,59]. In most of these conditions, iron overload toxicity is caused by increased body iron intake as a result of chronic red blood cell transfusions to treat the refractory anaemia and/or increased gastrointestinal iron absorption [60,61,62,63]. In such cases, iron overload leads to progressive multi-organ damage and an associated increase in the morbidity and mortality of the patients affected [64]. For example, the absence of iron chelation therapy in many regularly transfused TM patients in developing countries causes early fatalities, usually by the age of 20 years, mainly as a result of congestive cardiac failure associated with cardiac iron overload toxicity [64,65]. In contrast, following the use of iron chelation therapy, the survival of patients can increase; for example, the mean survival reported in the year 2000 of TM patients in the UK treated with DF has been estimated to be about 35 years [65]. More recently, the use of specific and effective iron chelation therapy protocols, including the use of L1/DF combination or, in some cases, L1 monotherapy, appears to cause an increase in the life expectancy and reduction in the morbidity of regularly transfused TM patients to levels approaching those of normal individuals [66,67,68,69,70].

In general, the presence of excess iron is a negative prognostic factor for all diseases, not only for cases involving specific organ damage such as in TM, but also for different cell types such as iron-loaded macrophages in cancer, as well as for subcellular organelles, such as excess iron deposition in mitochondria in Friedrich ataxia [57,59,62,71,72,73,74,75,76,77,78,79]. Usually, the susceptibility of each organ, cell, and subcellular organelle to iron toxicity is different and, in most cases, the level of toxicity is directly related to the level of excess iron load [71,80,81,82,83,84,85,86,87].

In relation to iron chelating drugs availability worldwide, all three drugs (DF, L1, and DFRA) are now classified in the generic class and also belong to the orphan drug category. Deferoxamine has been used parenterally for the treatment of iron overload conditions for more than 60 years [88,89]. Similarly, oral L1 has been used in India since 1995, in Europe and in other countries since 1999, and in the USA since 2011 [90]. Deferasirox is a relatively new oral drug and is very expensive, which has been registered worldwide since 2005, and the patent expired in 2017 [91].

The general properties of L1, DF, DFRA, and other chelators, including iron-binding, chemical, biochemical, pharmacological, toxicological, and clinical aspects, as well as other effects such as organ targeting and the level of iron excretion caused by each drug, have been previously reviewed (Figure 1) [10,33,90,92]. In general, the variable mode of action and of the overall pharmacological effects observed for each of the chelating drugs is a reflection of the differences in the drugs’ physicochemical and other molecular properties (Table 1). This variation is characteristic of each drug and has been shown in many in vitro, in vivo, and clinical studies.

**Figure 2:**
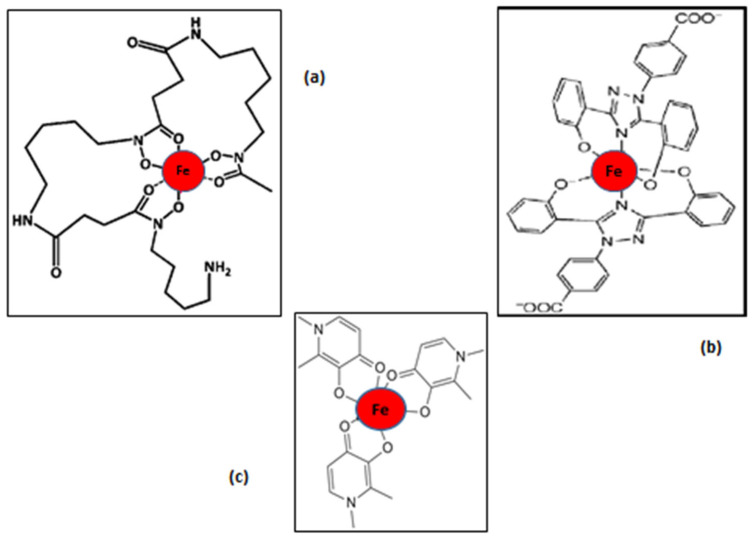
A diagram depicting the iron complexes of the iron chelating drugs deferoxamine, deferiprone, and deferasirox. Deferoxamine forms a 1:1 molar ratio stoichiometry complex with iron (Fe) (**a**), deferasirox forms a 2:1 molar ratio stoichiometry complex with iron (Fe) (**b**), and deferiprone forms a 3:1 molar ratio stoichiometry complex with iron (Fe) (**c**). All chelating drug iron complexes have an octahedral structure with iron in the centre, depicted as a red sphere.

For example, L1 is a bidentate chelator forming a 3L1:1Fe ratio stoichiometry complex, DFRA is a tridentate chelator forming a 2DFRA:1Fe ratio complex, and DF is a hexadentate chelator forming a 1DF:1Fe ratio complex at physiological pH (Figure 2) [10,33,90,92].

Many other parameters and factors are known to affect the iron chelating and redox activity properties of the chelating drugs, including, for example, the accessibility and presence of effective therapeutic concentrations of the drug at the oxidative damage target site, such as an affected organ or cell type. Similarly, differences in each individual patient case, such as drug absorption, distribution, metabolism, elimination and toxicity (ADMET) parameters, drug posology, timing and route of drug administration, drug interactions, underline disease, and other factors, can all affect the efficacy and toxicity of a chelating drug, as well as the overall outcome of the chelation treatment [44,52,93]. For example, the estimation of the lipid/water partition coefficient of the chelators and their iron complexes have shown that both L1 and DF are highly hydrophilic, whereas DFRA is highly lipophilic (Figure 1 and Figure 2) (Table 1). These physicochemical differences appear to affect the rate of transfer of iron from the iron complexes across the cell membrane of various cell types, as well as to cause variable effects in the extracellular and intracellular iron metabolic pathways of these cells [33,52]. Similarly, in relation to iron elimination, L1 appears to cause an increase in urinary iron excretion, DFRA in faecal iron excretion, and DF, mostly urinary but also some faecal iron excretion in iron-loaded patients (Table 1) [33,88,92].

### 3.2. Toxicity Limitations in the Use of Deferiprone, Deferoxamine, and Deferasirox

Despite the wide use of L1, DF, and DFRA in different diseases of iron overload, there is a limitation in their use in non-iron-loading conditions due to serious toxic side effects. In particular, major toxic side effects have been reported during the clinical use of both DF and DFRA in non-iron-loaded categories of patients. Furthermore, the administration of both DF and DFRA is not encouraged even for TM and other iron-loaded patients with serum ferritin lower than 0.5 mg/L [94,95].

Different toxic side effects have been reported for each of the iron chelating drugs in various categories of patients. For example, serious toxicities have been reported in preliminary studies in different non-iron-loaded categories of patients treated with DFRA, which, however, are not so frequently reported in iron-loaded TM patients [96]. The toxic side effects include renal, liver, and bone marrow failure and agranulocytosis, as well as other renal toxicities, skin rashes, and gastric intolerance [96,97,98,99,100,101,102,103]. In particular, kidney function is regularly monitored in TM patients treated with DFRA, and withdrawal of the drug is recommended for patients with a persistent rise in serum creatinine levels [95].

Similar limitations and restrictions as those reported for DFRA are also generally applied in the use of DF in non-heavily iron-loaded TM patients or other categories of patients with normal iron stores. In this context, and despite the fact that the incidence of serious toxicity is much lower in the case of DF than DFRA, the use of DF is not generally recommended in TM and other patients with low iron stores due to toxicity implications. Cases of mucormycosis, acute respiratory distress syndrome, and *Yersinia enterocolitica* are among the general serious toxicities reported in different categories of patients during the use of DF. In addition, auditory and ocular toxicity has also been reported in non-heavily iron-loaded TM patients using DF [90,104,105,106,107].

Several toxic side effects have also been reported during the use of L1 in TM patients and patients with normal iron stores. In the case of L1, the most serious toxic side effects appear to be those of agranulocytosis (1% >) and neutropenia (5% >) [92,108,109]. Both of these toxicities appear to be reversible. In this context, weekly or fortnightly mandatory blood count monitoring is recommended for prophylaxis for all patients using L1. Several other, less serious toxic side effects caused by L1 include gastric intolerance, joint/musculoskeletal pains, and zinc deficiency [92,110,111,112,113].

It appears that there is a variation between the iron chelating drugs not only in physicochemical and pharmacological properties, but also in their toxic side effects. In this context, the general rate of morbidity and mortality for each chelating drug in TM and other categories of patients is different, and also the target organ of toxicity varies in each case [92]. Furthermore, it also appears that the iron complex of chelating drugs is less toxic than the non-iron-bound chelator in all three cases [Figure 1 and Figure 2]. Similarly, there are no major toxicity reports and drug interactions of the chelating drugs with other drugs, which are used for the treatment of other co-morbidities in TM and other categories of patients with normal iron stores. However, toxicity vigilance, including the monitoring of drug interactions and also prophylactic measures, is generally needed for ensuring the safety of TM and other categories of patients treated with iron chelating and all other drugs due to polypharmacotherapy in many diseases, especially following the introduction of new drugs [114].

Overall, L1 appears to be the only chelating drug which is not restricted for use in patients with normal iron stores. In contrast, the limitations imposed on the use of DF and DFRA in TM and other patients with serum ferritin lower than 0.5 mg/L, which also includes all non-iron-loaded categories of patients, decrease the prospect of the wider use of DF and DFRA as chelator/antioxidant drugs in most diseases of FR pathology.

### 3.3. Repurposing of the Iron Chelating/Antioxidant Drug Deferiprone in Non-Iron-Loaded Diseases

The search for new pharmaceuticals for the treatment of many diseases affecting millions of people with no effective therapies, such as many types of cancer, Parkinson’s disease, and Alzheimer’s disease, as well as many orphan diseases such as malaria and other infectious diseases, is a priority for the patients affected, their families, and communities and also for world public health in general [50]. One of the most promising categories of approved drugs for repurposing and use in many of these diseases is chelating drugs, especially the iron chelating/antioxidant drug L1 (Figure 1) [50,96,115].

The drug repurposing efforts for the use of L1 in diseases other than transfusional iron overload, began within a few years from the initiation of clinical trials with L1 in iron-loaded TM and other transfused patients in the late 1980’s, and still continues to present times, involving many different categories of patients with normal iron stores (Table 2) [116,117,118,119,120]. The initial clinical studies in non-iron-loaded patients included anaemic rheumatoid arthritis and haemodialysis patients [116,117,119]. The repurposing of L1 in many other new categories of non-iron-loaded patients was originally proposed in 2003 and involved different abnormalities of iron metabolism, iron, and other metal toxicity, FR pathology, cancer, infectious and other diseases, etc. [121]. The criteria for selecting L1 for clinical studies in these different non-iron-loaded categories of patients were based on L1’s overall safety and efficacy potential, a risk/benefit assessment, preclinical studies and preliminary clinical findings in some cases and also the overall effects in iron-loaded patients [92]. Most importantly, the required background information on safety and efficacy was obtained from many preclinical studies involving many in vitro, cell, and animal findings in five different animal species, where some interspecies differences in relation to iron chelation were also observed [115,121,122].

Despite the encouraging results in preclinical studies, the repurposing of L1 in non-iron-loaded conditions was mostly based on the safety and efficacy of L1 in many iron-loaded categories of patients with various underlying conditions and different drug treatments (e.g., for diabetes, osteoporosis, hormonal complications, etc.) [114]. The different categories of iron-loaded patients treated with L1 in addition to TM were beta-thalassemia intermedia, HbE beta-thalassemia, HbS beta-thalassemia, sickle cell anaemia, myelodysplastic syndrome, aplastic anaemia, Fanconi’s anaemia, Blackfan-Diamond anaemia, pyruvate kinase deficiency, idiopathic hemochromatosis, iron overload in hemodialysis, juvenile hemochromatosis, etc. [92,115].

**Figure 3:**
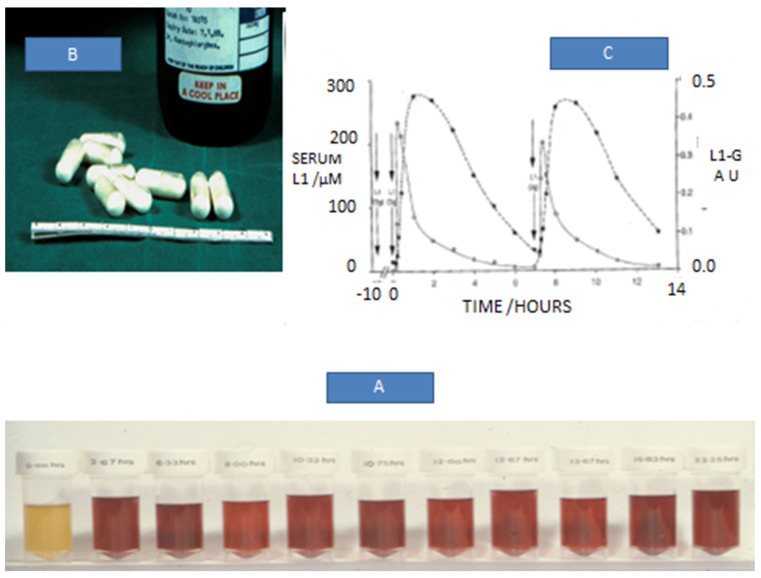
Historical photographs in relation to the first clinical trials of the first oral iron chelating drug deferiprone in London, UK, during the period 1987–1990. (**A**)—The first photograph indicates the maximum daily urinary iron excretion levels ever recorded using deferiprone in an iron-loaded thalassaemia patient. It shows sequential time urinary excretion samples over 24 h following the oral administration of deferiprone. The colour of the urine prior to deferiprone administration is yellow, then orange/red, similar to the iron complex of deferiprone. The total dose of deferiprone (16 g) was administered at five divided doses of 2 g and two doses of 3 g. The total 24 h urinary iron excretion was 325 mg. (**B**)—The prototype gelatine capsule formulation containing 0.5 g white solid of deferiprone, which was prepared during the first clinical trials in iron-loaded patients in London, UK, in 1987. (**C**)—Pharmacokinetic profile of deferiprone (L1) and its glucuronide metabolite conjugate (L1-G) following repeated oral administration of 3 g of deferiprone at −10, 0, and 7 h (horizontal axis). The left-hand side vertical axis shows the time profile of the serum concentration (μΜ) of L1 (solid line). The right-hand side vertical axis shows the time profile of the serum concentration in arbitrary absorbance units (AU) of (L1-G), the glucuronide metabolite conjugate of L1 (dotted line).

A further important finding for increasing the prospects for the use of L1 in non-iron-loaded categories of patients was the achievement of normal iron stores in ex-iron-loaded TM patients using L1 and L1/DF combination therapies, and also the maintenance of normal iron levels using L1 monotherapy for more than 100 patients’ years (Table 2) [123,124,125,126]. The achievement and maintenance of normal iron stores in TM patients was based on personalised drafted L1 dose protocols and regular body iron load assessment monitoring. This new approach signalled a new era in personalised medicine with the complete treatment of transfusional iron overload in TM patients using effective and safe chelation therapy protocols (Figure 3) [115,123,124,125,126]. The diagnostic criteria used for monitoring the gradual reduction of the body iron stores, as well as the characterisation of the maintenance of normal iron stores in ex-iron-loaded TM patients, were based on the reduction and maintenance of normal serum ferritin levels and also liver and cardiac magnetic resonance imaging (MRI) T2* signal intensity levels. The MRI T2* method has been used for estimating the iron deposition levels in the heart, liver, and other organs [127,128,129].

Organ damage and organ functioning complications in the heart, liver, pancreas, joints, etc., have been shown in many clinical studies of iron overload toxicity in TM and other similar iron-loaded conditions [114]. The level of toxicity in each of these cases was related to the level of iron overload, which was detected by the MRI T2* and other diagnostic techniques [127,128,129]. However, in most cases of iron toxicity and tissue damage identified in many non-iron-loaded categories of patients, the cause of the toxicity appears to be related to the presence of focal iron load deposits, which could be detected by the MRI T2* diagnostic technique. In contrast to TM and other iron-loaded patients, the serum ferritin levels of non-iron-loaded patients with focal iron deposition appear to be in the normal physiological range, reflecting the normal body iron store levels of this category of patients [79,82,83,84,85,86].

Focal iron load deposits in the brain with increased MRI T2* signal intensity have been detected in many neurodegenerative diseases, including Alzheimer’s disease, Parkinson’s disease, Friedreich’s ataxia, and neurodegeneration with brain iron accumulation (NBIA) [79,82,83,84,85,86,130,131]. It is interesting that at least fifteen diseases with NBIA have been characterised due to iron deposition in the globus pallidus and the substantia nigra parts of the brain [130]. Several other forms of toxic iron, such as toxic labile iron forms, have also been characterised and implicated in many other diseases of FR pathology (e.g., in diabetic and non-diabetic glomerular disease, ischaemic reperfusion injury, rhabdomyolysis, etc.) [132,133,134].

The therapeutic effects of L1 in different categories of non-iron-loaded patients have been examined in many clinical trials, where significant clinical improvements have been noted, especially when effective posology and appropriate diagnostic monitoring have been used (Figure 3) (Table 2). The majority of the non-iron-loaded conditions targeted by L1 were associated with neurodegenerative diseases, considering that L1 is the only one of the three iron chelating drugs that can cross the blood-brain barrier.

There have been many clinical trials reporting clinical improvements in the use of L1 in neurodegenerative diseases. For example, in a six-month duration study in Friedreich’s ataxia patients using L1 at 20–30 mg/kg/day, a reduction of excess iron deposition in the brain was observed, which was characterised by the MRI T2* diagnostic technique. The reduction of iron deposits in Friedreich’s ataxia patients was concomitant with a reduction in neuropathy and ataxic gait and without apparent serious toxicity, haematological, or neurological side effects (Table 2) [135]. Similarly, reduction of iron load in the basal ganglia, which was also characterised by MRI T2*, and a trend of slowing of disease progression has also been shown in patients with neurodegeneration with brain iron accumulation (NBIA) [136,137,138,139,140]. Furthermore, similar observations were also reported in Parkinson’s disease patients using L1, where slowing down of disease progression and improved motor function were shown in some patients [141]. However, in other clinical trials, the administration of L1 at single or repeated very low doses, such as that of 15 mg/kg/day, was mostly ineffective with disappointing results in both Parkinson’s and Alzheimer’s disease patients [141,142,143,144]. The findings were also questioned because of insufficient monitoring of other parameters in addition to low posology, such as the lack of pharmacokinetic and ferrikinetic data (Table 2) [144,145]. Several clinical trials using L1, involving different categories of neurodegenerative and other diseases, are currently in progress.

The encouraging results of in vitro, in vivo, and clinical trial studies on the safety and efficacy of L1, as well as its daily use in the last 30 years in thousands of TM and non-iron-loaded categories of patients worldwide confirms L1’s high safety record and its suitability to be used as a universal repurposed chelator/antioxidant drug in all diseases related to FR pathology and in particular for diseases where no effective treatments are available (Figure 3) [50,115,146,147,148]. Further confirmation of the chelator/antioxidant effects and safety of L1 has been shown in clinical trials involving many other categories of patients in addition to neurodegenerative diseases, including cardiovascular, renal, infectious diseases, cancer, AIDS, and ageing (Table 2) [50,115,149,150,151].

It should be noted that the focus and diagnostic criteria used for monitoring L1 in almost all the clinical studies of both the iron-loaded and non-iron-loaded categories of diseases were based on safety parameters, and also the therapeutic outcome in patients of each clinical condition. Furthermore, comparison to other drug treatments under the same conditions was carried out, if any such treatments were available at the time of the clinical study.

It is interesting that in most clinical trials, the antioxidant potential of L1 or other drugs is not usually monitored or considered part of the clinical therapeutic protocol. For example, in iron chelating drug testing in iron overload, body iron elimination and removal of excess iron from organs are mostly monitored because such effects decrease or eliminate the potential of molecular, cellular, and tissue damage in TM and other iron-loaded conditions (Figure 3). In particular, it has been shown in many and extensive clinical investigations that L1 can remove all excess toxic iron from the heart of TM patients, which is the target organ of iron toxicity and the main cause of mortality in TM [64,65]. The iron removal treatment by L1 resulted in the concomitant progressive improvement of cardiac function and prolonged survival of TM patients [66,67,68,69,70,123,124,125,126,127,129].

Further investigations of regular cardiac monitoring have also shown that the long-term use of L1 significantly enhanced left-ventricular ejection fraction (LVEF) and improved the antioxidant status of the patients [152,153,154,155,156]. Similar cardiac improvements following L1 therapy have also been observed in other categories of iron-loaded patients with cardiac complications [157,158,159,160]. Studies at the cellular level have also suggested that improvement of the LVEF was related to the antioxidant effects of L1 on endothelial cells [161,162]. Several other improvements in the antioxidant status, such as increases in reduced glutathione levels and also in cellular function, were observed in the red blood cells of iron-loaded patients and also in animals treated with L1 [156,163,164,165]. The antioxidant effects of L1 have also been shown in many other in vitro and in vivo experimental models of oxidative damage [51,52,117,166,167].

The safety and other parameters associated with the iron chelating/antioxidant pharmacological activity of L1, and also its wide distribution in cells and organs, suggest that L1 could be applied as a universal chelator-antioxidant drug, primarily targeting toxic iron forms involved in the Fenton reaction and other pathways leading to oxidative damage and related diseases of FR pathology (Figure 1, Figure 2 and Figure 3). Furthermore, it also appears that L1 could be used as a general antioxidant drug for the prevention, delay, or reversal of OST-related tissue damage caused by iron and copper catalytic activity. In particular, L1 could also be used as a general inhibitor or modulator of ferroptosis, which is observed in almost all diseases. In this context, increasing numbers of recent studies have shown that L1 could inhibit ferroptosis in neuronal cells associated to neurodegenerative diseases including Alzheimer’s and Parkinson’s diseases [168,169,170,171], in retinal cell damage [172,173,174,175], infections [176,177], cardiac, liver, kidney and other organ damage [178,179,180,181,182], anticancer drug toxicity [183], colorectal cancer and hepatocellular carcinoma [184,185], environmental damage [186], and many other conditions [187,188,189,190].

Further clinical trials are needed for the evaluation of L1 as a universal iron and copper chelator-antioxidant drug in diseases associated with FR pathology and ferroptosis, including combinations with other drugs, iron and copper chelator drugs, and other natural or synthetic antioxidants.

### 3.4. The Repurposing Prospects of Deferoxamine, Deferasirox, and EDTA as Antioxidant Drugs

A number of other chelating drugs in addition to L1, including mainly DF, DFRA, and EDTA, have also been tested for repurposing purposes in several non-iron-loaded diseases, and also for antioxidant activity (Figure 1). The protocols used for these three iron chelators in each non-iron-loaded disease were, in most cases, designed to limit the prospect of serious toxicity under specific conditions and, in most cases, included protocols of low drug doses for short-term or periodic administration. There is still no consensus in the evaluation of each of these three chelating drugs, and, in most cases, different protocols, including drug posology and formulations, have been used for each targeted disease. As in the case of L1, the diseases selected for clinical testing had no known effective treatments with other drugs.

Deferoxamine is the oldest iron chelating drug, which is not orally effective and is mostly administered subcutaneously (SC) or intravenously (IV) (Figure 1). Despite the difficulties with the parenteral administration, there have been many approaches for the repurposing of DF in many clinical conditions in addition to iron overload, including the design of different formulations, routes of administration, and posology. In particular, several clinical trials have been reported in the last 50 years using DF in relation to its antioxidant activity, including ischaemia/reperfusion injury, intracerebral haemorrhage, organ transplantation, and increase in angiogenesis, skin damage, wound healing, cancer, and neurodegenerative diseases. In almost all these diseases, the major target for DF was iron associated with OST identified in specific cells of different organs or in relation to metabolic or other forms of iron toxicity.

In particular, encouraging findings related to the efforts for repurposing DF in non-iron-loaded diseases were reported in many clinical trials using a variable selection of dose protocols and drug formulations. For example, in a clinical study with patients undergoing coronary artery bypass grafting, the IV infusion of 4 g of DF over 8 h caused amelioration of FR/ROS production and protection of the myocardium against ischaemia/reperfusion injury [191]. This DF protocol was more successful, especially in patients with lower left-ventricular ejection fraction (LVEF) [191]. Similar antioxidant and other beneficial effects were observed in alleviating ischaemia/reperfusion injury in cardiopulmonary bypass patients in comparison to patients receiving standard care and also in phase I randomised clinical trials using a combination of DF with ascorbic acid and N-acetylcysteine (Figure 1) [192,193].

Another major area of increased prospects for the repurposed clinical use of DF is traumatic brain injury, intracerebral haemorrhage, and ischaemic stroke, where a number of clinical trials have shown encouraging results [194]. In a series of studies by an intracerebral haemorrhage DF trial group, a follow-up of 146 patients in a phase II clinical study for 6 months has shown a significant improvement over a control group using DF at 32 mg/kg/day IV infusions for 3 consecutive days within 24 h of haemorrhage onset [195,196]. In another phase II clinical study in ischaemic stroke patients by another group of investigators, DF was administered through IV as a bolus (10 mk/kg), followed by 72 h continuous IV infusion of three escalating doses (40–60 mg/kg/day) [197]. Improvements were noted in 31% of the placebo patient group versus 50–58% in the DF-treated patient group [197]. Similar approaches in the use of DF in ischaemia/reperfusion injury in liver and other organs, and also in organ transplantation conditions, tissue regeneration via promotion of angiogenesis, retinal damage, wound healing, etc., have also been reported [198,199,200,201,202,203].

The disadvantages of oral inactivity, rapid clearance from blood, and inability to cross the blood-brain barrier by DF prompted investigations for its administration via different routes and formulations, for example, as a suppository or intranasal preparations, not only for iron-loaded patients, but also for other conditions [204,205,206]. In this context, there is an increasing interest in the intranasal administration of DF for the treatment of neurodegenerative and other conditions affecting the brain, including Alzheimer’s and Parkinson’s diseases [206,207,208]. It appears, in general, that the intranasal administration of DF bypasses the blood-brain barrier and allows targeting of the central nervous system, which is crucial for increasing the prospects in the treatment of neurodegenerative diseases [208,209]. However, in much earlier randomised controlled studies, DF has also been shown to slow the clinical progression of the dementia associated with Alzheimer’s disease, even when it was administered intramuscularly (125 mg twice daily, 5 days per week) for 24 months [210].

The antioxidant potential of DF has also been shown in other categories of patients, including rheumatoid arthritis, cancer, and skin protection in cosmetics, where further investigations were suggested for maximising the efficacy and minimising the toxicity of DF [211,212,213,214,215,216,217].

The iron chelating/antioxidant potential of DF has also been shown in many experimental disease models through the iron-binding effects and association with ferroptosis inhibition [35]. The inhibition of ferroptosis by DF involved many disease models of organ damage, including brain damage and neurodegeneration [218,219,220,221], other organ damage [222,223,224,225], cancer [226,227,228], environmental and drug toxins, etc. [229,230,231].

In contrast to DF, limited were the efforts for the repurposing of DFRA as an antioxidant drug, considering that some antioxidant potential was shown in vitro and in relation to ferroptosis, but also during chelation treatment in iron-overloaded diseases. In particular, decreases with oxidative stress parameters have been shown during randomised clinical trials of DFRA involving iron-loaded TM, haemodialysis, sickle cell anaemia, and myelodysplasia patients [232,233,234].

Different drug protocols were used in the clinical trials of DFRA in the various groups of patients. In one study involving 49 TM patients, DFRA was administered at 18.6 ± 7.6 mg/kg/day for up to a year and compared to DF at 46.8 ± 8.8 mg/kg/day, resulting in a significant decline in iron-load and the oxidative-stress marker malondialdehyde with 22% per year reduction for DFRA and 28% per year reduction for DF [232]. Similarly, oxidative-stress markers were monitored in a different comparative study involving a control group of 30 normal volunteers, a matched group of 30 TM patients treated with DFRA (20–40 mg/day), and a group of 30 TM patients treated with IV DF (20–50 mg/day). The total antioxidant capacity (TAOC) was reported to be higher in the control group, followed by the DF group, and lowest with DFRA, while malonaldehyde production was in the reverse order [235].

Similar results were obtained during an investigation into the antioxidant potential of DFRA in a clinical trial of a 388-day duration involving iron-loaded sickle cell anaemia patients [236]. In this case, three groups were compared: a group of 15 patients receiving DFRA, a second group of 10 patients receiving a combination of DFRA and hydroxyurea, and a third group of 15 patients receiving folic acid. An increase in trolox-equivalent capacity and a decrease in thiobarbituric acid reactive substances in the DFRA-treated group were observed. Similar changes were observed in the combination of DFRA and hydroxyurea group, but no changes in the folic acid group at the end of the study [236].

In another comparative 6-month study involving iron-loaded haemodialysis patients, 54 patients received DFRA (15 mg/kg/day) compared to 50 patients not receiving chelation. Significant reduction of serum ferritin and thiobarbituric acid reactive substances in the DFRA-treated group was reported in comparison to the non-chelated group [233].

The effect of DFRA (20 mg/kg/day) on labile iron and oxidative stress was also investigated in a clinical study of 3 months following 19 iron-loaded myelodysplasia patients. The reduction in ROS and lipid peroxidation of red blood cells and an increase in GSH in red blood cells, platelets, and polymorphonuclear leukocytes were observed, which were associated with the chelation and removal of intracellular and extracellular toxic labile iron by DFRA [234]. Two deaths and other toxic side effects were observed in 10 of the 19 myelodysplasia patients [234]. Many other toxic side effects have also been reported in many other categories of patients, limiting the prospects of repurposing DFRA in non-iron-loaded conditions [97,98,99,100,101,102,103,237,238,239,240,241].

Inhibition of ferroptosis has also been shown in some experimental models of different diseases, despite the fact that the iron chelating and antioxidant potential of DFRA is much lower than that of L1 and DF [242,243,244,245,246,247,248,249].

Several other chelating drugs with high iron-binding potential, including EDTA and DTPA, are also known to affect iron and other metal excretion, as well as associated iron metabolic and redox pathways [250,251]. In particular, one of the recent efforts in drug repurposing was related to the chelating drug EDTA, which was originally approved for the detoxification of lead (Figure 1). In general, lead and other heavy metal toxicity are considered to be partly associated with an increase in FR/ROS damage in many clinical conditions [18,19,20,252,253,254,255]. EDTA has been used as an alternative treatment for atherosclerosis and many conditions associated with heavy metal toxicity in alternative medicine clinics worldwide in the last 50 years [251,256,257]. In most long-term clinical studies and alternative medicine uses, EDTA is usually administered through IV as a disodium formulation or calcium disodium formulation about once every two weeks. After EDTA administration, metal ion excretion is usually measured in a 24 h urine collection to ensure, among other things, that there is essential metal ion maintenance and avoidance of associated metal deficiency toxicity.

Following many controversies regarding the wide use of EDTA in alternative medicine clinics, a number of clinical trials have been carried out in the last few years in conjunction with the USA health authorities to assess the effect of the disodium EDTA chelation regimen on cardiovascular events and diabetes, in patients with previous myocardial infarction [257,258]. In one study, 839 patients received 30 IV infusions weekly of a 500-mL solution containing mainly 3 g of disodium EDTA and 7 g of ascorbic acid, compared to a placebo group (n = 869). A further 10 infusions over a 2 to 8 week duration followed. A modest reduction of the risk of adverse cardiovascular outcomes was observed in the EDTA group, which, however, was not of a sufficient level to suggest the routine use of EDTA chelation therapy for treatment of all patients who have had a myocardial infarction [257]. Similarly, in a further evaluation of the clinical trial results, it was also suggested that a high dose of multivitamins alone, or in conjunction with EDTA, did not cause a reduction of cardiovascular events in patients with chronic coronary disease, diabetes, and a previous myocardial infarction [258].

Many other clinical studies were carried out using EDTA, showing improvements in some patients with different disease categories, especially those involving heavy and other toxic metals [251,259,260,261]. However, pro-oxidant effects, fatal and other toxicities, have also been observed in patients treated with EDTA, suggesting that continuous patient monitoring is important for preventing or reducing adverse effects related to EDTA treatment [262,263,264,265,266,267].

Overall, preclinical studies and clinical trials have been carried out with DF, DFRA, and EDTA in different models of oxidative damage with encouraging findings, and increased prospects for their repurposing in some diseases associated with FR pathology and ferroptosis. However, further studies are required for optimising the use and reducing the toxicity of these drugs as antioxidants in different non-iron-loaded diseases and also for other therapeutic applications. These include the identification of specific targets related to OST, the selection of therapeutically effective and non-toxic drug posology, and the characteristics, as well as the categories of patients who can mostly benefit from their repurposing as antioxidant drugs and/or as therapeutics. Furthermore, a risk/benefit assessment is required in the selection of each chelating antioxidant drug case, as well as the advantages of their application in comparison to existing therapies in each disease.

## 4. Iron-Binding Drugs, Pro-Drugs, and Drug Metabolites with Antioxidant Properties

Many drugs, in addition to L1, DF, DFRA, and EDTA, have been reported to have iron chelating properties and to be involved in redox effects, including antioxidant activity (Figure 1 and Figure 2). The antioxidant potential of these drugs, and also their possible clinical application in conditions involving OST damage and diseases associated with ferroptosis, have not yet been fully investigated. Similarly, several cases of pro-drugs and drug metabolites with iron-binding and antioxidant properties have also been reported and may potentially further be developed for clinical use for antioxidant activity in conditions involving FR toxicity. In this context, some promising examples of iron-binding drugs, such as N-acetylcysteine, aspirin, and dexrazoxane, will be discussed, emphasising their antioxidant application in diseases of FR pathology.

### 4.1. The Antioxidant Clinical Effects of N-Acetylcysteine and Its Iron-Binding Properties

One of the most widely used repurposed drugs, which has been tested for antioxidant activity in many clinical conditions of OST damage, is N-acetylcysteine (NAC) (Figure 1). Initially, this generic drug was approved about 50 years ago by the FDA as an antidote for the treatment of acetaminophen (paracetamol) overdose toxicity, where liver glutathione has been shown to be depleted and to cause liver damage. N-acetylcysteine is also currently used clinically for mucolytic activity, mostly in bronchopulmonary diseases [268].

N-acetylcysteine is a low molecular weight, charged, hydrophilic molecule with low permeability of biological membranes, including the blood-brain barrier (Figure 1). It can be administered orally or intravenously. It is poorly absorbed orally (4–9%), metabolised mostly in the liver, and about 30% is excreted in the urine. The plasma half-life of oral NAC is about 5–6 h [269].

The main mode of the antioxidant activity of NAC is based on the increase of the intracellular concentration of cysteine, a precursor of the natural antioxidant glutathione, which is required for the neutralisation of the increased production of hydrogen peroxide during oxidative stress. The thiol group in NAC also appears to be involved in different biological activities, including the inhibition of oxygen and nitrogen FR production, interactions with proteins containing thiol groups, and the formation of complexes with iron and other metal ions [269].

Many clinical trials have been carried out using NAC as an antioxidant either as monotherapy or in combination therapies in different categories of patients. In most cases, the antioxidant activity of NAC has been tested using therapeutic protocols of oral or intravenous administration. Despite the generally acceptable safety profile regarding clinical interventions using NAC, the overall therapeutic outcome in most cases was not satisfactory, with mostly negative or equivocal findings. However, benefits or improvement of therapeutic indices and antioxidant status were reported in a few studies and in some categories of patients. Similarly, the overall toxicity with different NAC treatment protocols was low, despite the fact that in some cases, fatal and other permanent or serious toxic side effects have been reported, especially in NAC overdose [270]. Common toxic side effects of NAC include nausea, vomiting, rhinorrhea, rash, urticaria, pruritus, bronchospasm, and tachycardia [270].

Different dose protocols were used in clinical trials to assess the efficacy and safety of NAC. For example, an IV infusion of 1–16 h duration of NAC at 50–150 mg/kg/day was used in one study for the optimisation of treatment against acetaminophen overdose toxicity [270]. In another phase I study in traumatic brain injury in children, NAC doses of 140 and 70 mg/kg/day were also administered by IV infusion in combination with probenecid at 25 and 10 mg/kg/day. In the latter study, both NAC and probenecid were detected and measured in the cerebrospinal fluid of the patients. However, the therapeutic outcome results of the NAC and probenecid combination were not different from a placebo group [271]. Similar results were obtained when oral NAC at 2.7 g/day for 6 months was used for the treatment of early psychosis patients. In this case, the results have shown an improvement in the antioxidant biomarker parameters, but not a therapeutic outcome better than the placebo group [272].

N-acetylcysteine has been tested in many other different categories of patients using various dose protocols and duration of studies, with no conclusive positive outcome in the pathology of each patient category tested. For example, in a meta-analysis report of acute respiratory distress syndrome, the IV infusions of NAC at 40–210 mg/kg/day for 3–10 days suggested that clinical benefits were limited [273]. Similar findings and conclusions were drawn from a meta-analysis of 29 studies involving NAC and other antioxidants in women with pre-eclampsia and perinatal death [274]. No significant changes were also observed in the pathology of kidney disease patients, including contrast-induced nephropathy, acute kidney injury, peritoneal dialysis with chronic kidney disease, and kidney transplant patients who received NAC and other antioxidants. There was, however, a positive response reported for some patients in the last group [275].

The efforts to combine NAC with other drugs as adjuvant therapy have recently attracted some interest in conditions known to have increased OST damage. In some of these trials (e.g., pulmonary tuberculosis, ischaemic stroke, and psychotic disorders), NAC has shown no effect [276,277,278]. However, positive outcomes have been shown in patients with steatotic liver disease, atrial fibrillation after coronary artery bypass graft surgery, in prophylaxis ventilator-associated pneumonia, and in sepsis in intensive care units [279,280,281,282]. Similarly, other reports suggest that the prospect of use of NAC in neurological diseases also appears to be encouraging [283].

A major area for the clinical testing of antioxidants, including NAC, is cancer. This is particularly important considering that OST has been identified to play a major role in different stages of cancer, including initiation, progression, and metastasis. Furthermore, the prospects of chemoprevention in cancer using antioxidants are widely discussed in the medical literature and the mass media. In this context, many clinical studies have been carried out using NAC and other antioxidants in different categories of cancer patients (e.g., head and neck cancer, lung cancer, melanoma, hepatocellular carcinoma, breast cancer, prostate cancer, etc.) [284,285,286,287].

Different NAC dose protocols, combination therapies, and targets were selected and used in the studies involving cancer patients. For example, NAC was administered to 12 early diagnosed breast cancer patients for 19 days prior to surgery, initially once a week at a dose of 150 mg/kg using IV infusion and 600 mg orally twice daily on the remaining days. This protocol appears to be safe, well-tolerated, and reduces the carcinoma cell proliferation rates in this cohort of cancer patients [288]. Many other cancer-related effects, such as mutagenesis, chemotherapy toxicities, and adjuvant therapies, have also been studied using NAC [289,290]. The results were not satisfactory in most cancer studies, despite the fact that some antioxidant and therapeutic parameters were improved [287]. Furthermore, posology appears to be a critical factor for the assessment of NAC, considering that in a monitoring study involving about 270,000 chronic hepatitis C patients, the use of higher doses of NAC appears to be more effective than lower doses in preventing hepatocellular carcinoma [284]. Positive results were also anticipated in a meta-analysis of 32 articles involving 2500 cancer patients in randomised clinical trials discussing the mitigation of toxicity in cancer chemotherapy by antioxidants, including NAC. In this case, it was suggested that antioxidant supplementation during cancer chemotherapy holds the potential for reducing dose-limiting toxicities [291]. Similarly, positive outcomes have been reported using NAC in hearing loss in children and other side effects of cisplatin toxicity [292,293].

The effects of NAC in the amelioration of iron toxicity have also been investigated in different categories of patients and under different conditions. In particular, some limited benefits were identified in clinical studies involving NAC in iron-loaded haemoglobinopathy patients with sickle cell disease and thalassaemia, as well as in the preservation of stored red blood cells [294,295,296]. Overall, more positive outcomes were reported in clinical trials in the use of NAC in thalassaemia patients in comparison to sickle cell disease patients [297,298].

N-acetylcysteine has also been reported to inhibit ferroptosis associated with different pathways and disease models of OST, which were related to many organs and various clinical conditions [299,300,301,302,303,304]. Furthermore, NAC has also been shown to inhibit ferroptosis in different cancer models [305,306,307,308].

The overall assessment of NAC in different clinical conditions appears, in general, to be positive with regard to safety, but not conclusive for its use as an antioxidant drug that can prevent the pathological implications in any specific disease. Further efforts for the development of NAC as a repurposed antioxidant drug are needed to comply with drug regulatory requirements in one or more diseases. In particular, further evaluation and optimisation of the therapeutic effects of NAC are necessary in each disease, including definition of the mode of action, target specificity, dose protocols, and duration of administration. Similarly, further evaluation and optimisation of the therapeutic effects of NAC is also needed following its co-administration with other antioxidants or drugs for the achievement of therapeutic goals and of maximum therapeutic outcome in each targeted disease.

### 4.2. Pro-Drugs and Drug Metabolites with Iron-Binding and Antioxidant Properties

The efficacy and safety of iron chelating drugs, nutraceuticals, and dietary molecules could be affected in their antioxidant capacity by many interactions, including those between them, for example, in competition for iron-binding, interactions with other metal ions, variable effects on different antioxidant targets, etc. Similarly, ADMET characteristics, metallomic, redoxomic, pharmacogenomics, proteomic, metabolomics, and other factors could also affect the efficacy and safety of each antioxidant within the concept of personalised medicine (Figure 3) [10,44,47,51,93,114].

**Figure 4:**
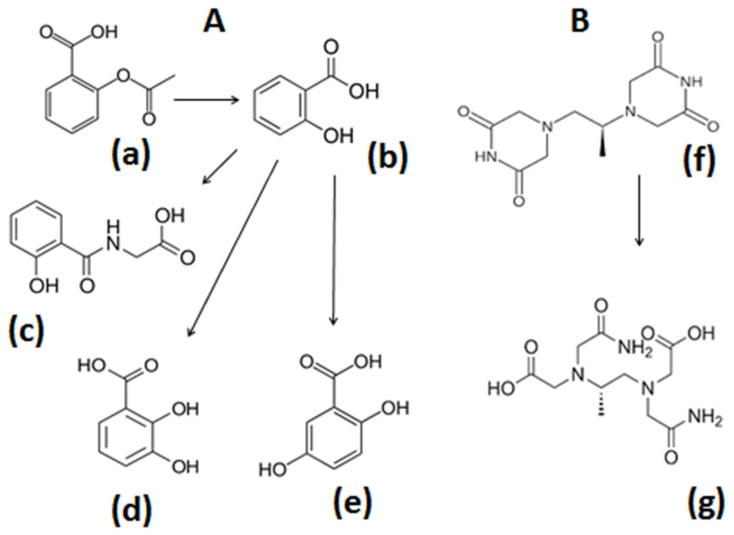
The biotransformation of the drugs aspirin and dexrazoxane into iron chelating metabolites. Aspirin (acetylsalicylic acid) (**a**) is metabolised to several metabolites, including salicylic acid (**b**), salicyluric acid (**c**), 2,5-dihydroxybenzoic acid (gentisic acid) (**d**), and 2,3-dihydroxybenzoic acid (**e**), all of which have iron-binding and antioxidant properties (**A**). Dexrazoxane (**f**) is biotransformed into ADR-925 (**g**), an EDTA-like iron chelating metabolite, which reduces anthracycline cardiotoxicity in cancer patients (**B**).

A new concept of antioxidant drug development is the activity and role of iron chelating drug metabolites. Recent studies have suggested that several drugs, pro-drugs and drug metabolites, appear to play an important role in the design of iron chelating/antioxidant strategies. In particular, the metabolism of DF, ascorbate, dexrazoxane, and aspirin appears to play an important clinical role in antioxidant activity in cancer and other conditions, especially through their iron chelating metabolites (Figure 4) [309,310,311,312,313,314,315,316,317].

Dexrazoxane is widely used in cancer patients against doxorubicin and other anthracycline drug cardiotoxicity [315,316,317,318]. In humans, dexrazoxane is metabolised to ADR-925, an EDTA-like iron chelating compound (Figure 4B). The cardioprotective effect of dexrazoxane is considered to partly involve the removal of iron by ADR-925 from the redox-active–iron complex of doxorubicin, which causes OST damage to cardiomyocytes (Figure 4B) [315,316,317]. However, despite the beneficial effects against anthracycline drug cardiotoxicity, the toxicity of dexrazoxane is also a limiting factor for its use in cancer patients [318,319,320]. It is interesting that L1 has also shown cardioprotective effects against doxorubicin toxicity in cell studies, and also in clinical studies in iron-loaded thalassaemia and other patients [152,153,154,155,161,162,321].

Aspirin is another example of a pro-drug which can be biotransformed into several iron chelating/antioxidant metabolites (Figure 4A). In particular, the four aspirin iron chelating metabolites salicylic acid, salicyluric acid, 2,5-dihydroxybenzoic acid, and 2,3-dihydroxybenzoic acid, which amount to 70% of the aspirin administered dose and have a much longer half-life than aspirin in blood and tissues, appear to play a major pharmacological role in the overall pharmacological activity of aspirin (Figure 4A) [312,313,314]. In this context, epidemiological studies have suggested that, following the administration of aspirin in elderly patients at 75–100 mg/day for longer than 5 years, 20–30% of patients had a lower risk of developing colorectal cancer, and about the same proportion had a greater risk of developing iron deficiency anaemia [314,322]. The latter is suspected to be due to an increase in iron excretion caused by aspirin’s chelating metabolites, especially in vegetarians, where low iron diets and low body iron intake are observed [314]. It has also been suggested that the reduction in cancer risk is likely to be due to the targeting by aspirin’s chelating metabolites of iron involved in FR damage, as well as iron toxins, in iron proteins, and associated metabolic pathways, including ferroptosis [322].

It should be noted that salicylic acid, salicyluric acid, 2,5-dihydroxybenzoic acid, and 2,3-dihydroxybenzoic acid are naturally occurring plant products with well-known iron-binding and antioxidant properties (Figure 4A) [314]. Overall, it seems that the daily use of low-dose aspirin for many years by the elderly population for prophylaxis, mainly against cardiovascular disease, is also acting as a chemopreventive and anticancer agent through aspirin’s chelating metabolites [322]. Many other mechanisms have also been suggested for the anticancer mechanisms of aspirin in different types of cancer [323,324,325,326,327,328,329,330]. Similarly, different mechanisms for the inhibition of ferroptosis by aspirin and its iron chelating/antioxidant metabolites have also been suggested [322,331,332,333,334,335].

Further investigations are needed for identifying pro-drugs, nutraceuticals, and phytochelators which are biotransformed into metabolites with iron chelating/antioxidant properties for targeting OST damage in different organs, and also for their wider development and application in medicine. Different targeting strategies could also be designed and developed using iron chelating/antioxidant pro-drugs and nutraceuticals for topical application, enterohepatic circulation, and other pathways of metabolism and excretion.

## 5. Future Prospects and Strategies in Antioxidant Therapeutics

No antioxidant drugs have yet been developed or prescribed in medicine, despite the hundreds of thousands of publications on antioxidants and the hundreds of clinical trials in different diseases of FR pathology, where, in many cases, improvements in different OST markers and other pathological parameters have been noted. At the same time, millions of people, including many different categories of patients, are using antioxidant nutraceuticals and traditional folk medicines, as well as EDTA in alternative medicine clinics every day, for chemoprevention or treatment (Figure 1) [48,49,251,257,258,259].

Ideally, effective antioxidant therapeutic strategies may involve the design of a multitarget drug for the effective and safe inhibition of oxidative damage through iron chelation, the enhancement of the antioxidant defences by neutralising FR, the triggering of increased production of endogenous antioxidants, and also the triggering of the direct or indirect activation of antioxidant mechanisms, as well as other factors. However, such approaches are difficult considering that, usually, different drug applications are needed for each disease and each target, and also that there is great variability in organ distribution and metabolism for each potential antioxidant drug, nutraceutical, or nutrient.

The possibility of antioxidant drug development by pharmaceutical companies is currently highly unlikely because it is very expensive and primarily based on commercial considerations, such as patent exclusivity and monopolies on sales. In this context, the interest of pharmaceutical companies in developing antioxidant drugs seems not to be an attractive proposition, mainly because of the lack of sufficient clinical evidence in common or orphan diseases and also questionable commercial benefits [50]. In addition, in many clinical studies using antioxidant drugs, the improvement of antioxidant biomarker parameters does not appear to be translated into therapeutic improvement or therapy in any disease. In this context, further investigations are needed to clarify the level of contribution of antioxidant therapies in the treatment of each disease.

Up until recently, most efforts for the development of antioxidant drugs were mainly based on individual academic initiatives and focused on generic drugs, nutraceuticals, and natural products with antioxidant properties. A new approach for the design and development of antioxidant pharmaceuticals for clinical use is to consider new strategies and the organisation of joint concerted efforts by interested groups, which will include arrangements for pre-clinical and clinical testing through the required regulatory route and submission of findings to the drug regulatory authorities for antioxidant drug approval [50]. Such regulatory processes may be successful, considering that academic drug development (e.g., in the cases of L1 and ferric maltol) was subsequently exploited by generic and small pharmaceutical companies, and the drugs eventually became commercially available based on emergency use and satisfaction of orphan drug requirements [90,115,336]. In particular, L1 was initially used in the UK, Europe, and the USA on a named patient permission, which was approved by hospital ethics committees or regulatory drug authorities, especially in cases where DF was contraindicated due to toxicity (Figure 3). Deferiprone was also first registered in India for the treatment of iron overload in TM due to the unaffordable expenses and toxicity associated with SC DF treatment, and also following confirmatory clinical trials related to L1’s efficacy and safety, which were controlled by the Indian drug regulatory authorities [90,115,336].

Similar approaches have been considered by the current use of L1 in the treatment of patients diagnosed with neurodegeneration caused by brain iron accumulation, since L1 is the only registered drug capable of preventing excess iron toxicity in the brain [136,137,138,139,140]. Similarly, ferric maltol, which was invented more than 40 years ago for the treatment of iron deficiency anaemia, was recently approved for the treatment of iron deficiency anaemia in inflammatory bowel disease patients, since all other iron formulations could not be tolerated by this category of patients [336,337]. Following this initial approval, ferric maltol is also currently prescribed for other categories of iron-deficient patients in addition to patients with inflammatory bowel disease [337]. In this context, similar approaches and strategies could be designed for the regulatory approval of promising antioxidant drugs by focusing on the treatment of identified and measurable toxicity of oxidative damage in specific clinical conditions.

**Figure 5:**
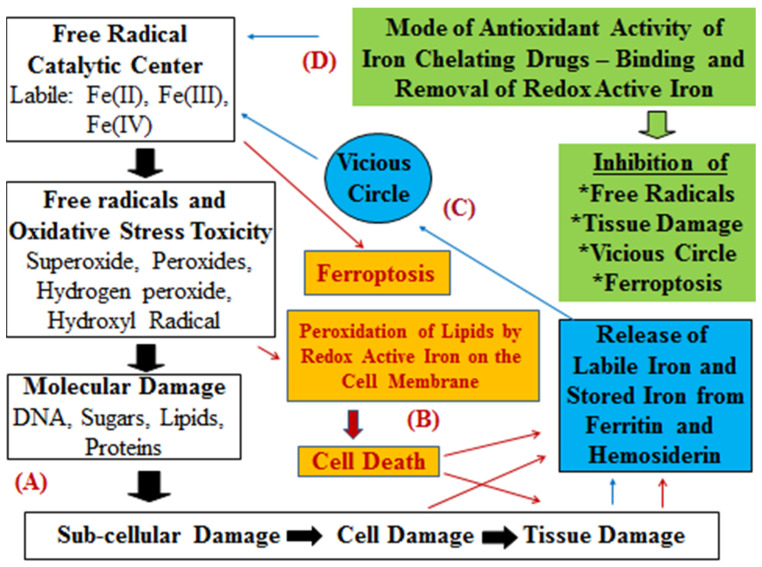
The potential targets of oxidative damage and the mode of action of iron chelating/antioxidant drugs for the inhibition of the catalytic role of iron in free radical pathology and ferroptosis. Iron catalyses the formation of free radicals; causing oxidative stress toxicity; leading to molecular, subcellular, cellular, and tissue damage (**A**). A similar pathway is followed in ferroptosis, where iron catalyses the formation of free radicals, causing lipid peroxidation on the cell membrane and programmed cell death (**B**). Cell and tissue damage and ferroptotic cell death cause the release of toxic iron, resulting in a vicious circle of free radical production and damage (**C**). The iron chelating drugs deferoxamine, deferiprone, and deferasirox act as antioxidants by binding and removing labile toxic iron, which catalyses free radical production (Fenton reaction), thus preventing tissue damage and ferroptosis (**D**). The asterisk (*) highlights the major mode of therapeutic action of iron chelating drugs in free radical pathology).

The diversity of clinical use of antioxidant drugs is also of great interest. For example, there are many variations in the strategy for the design of new antioxidant drugs for different uses. Some may depend on the duration of the oxidative damage and the antioxidant administration, for example, long-term administration in chemoprevention, short-term administration in ischaemia/reperfusion injury, and also other time periods depending on the cause of oxidative damage. Similarly, the strategy for the development of new drug antioxidants can range from antioxidant drugs with broad-spectrum activity to more specific antioxidant drug targeting at various levels, from molecular to tissue and organ targets (Figure 5). More effective antioxidant strategies could also be potentially developed involving therapeutic protocols of combinations with other antioxidants and also other drugs. This approach resembles iron chelation combination therapy, which is generally more effective than monotherapy [114,124,125,154,161].

Despite the optimistic prospects from some clinical trials, many limiting factors could undermine the efforts for the introduction and use of targeted antioxidant drugs in clinical practice. These may include the lack of convincing and reproducible results from randomised clinical trials, the lack of specificity and effective reversal of the oxidative toxicity damage by the antioxidant drug for the targeted disease or tissue affected, the selection of the wrong antioxidant drug posology for clinical testing, the risk/benefit assessment method, the advantages over other drugs, which may have already been used for treatment, etc. [50,142,143,144,145].

In the meantime, several other new strategies have recently emerged, which may also be considered for the development of new antioxidant drugs. These may involve the identification of pro-drugs, such as aspirin, which is biotransformed and partly forms iron chelating/antioxidant metabolites with possible clinical applications in antioxidant chemoprevention and other therapeutic programmes (Figure 4A) [314,315,316,317,318,319,320,321,322]. Future strategies for the development of a new class of antioxidant therapeutics for clinical use may also involve the development of iron chelating/antioxidants undergoing enterohepatic circulation, and also new, more effective formulations of known or new iron chelating/antioxidant drugs. Similarly, the targeting of many diseases associated with ferroptosis with iron chelating/antioxidant drugs is likely to increase the interest for the development of new related therapeutics for cancer and many other diseases [338] (Figure 5).

Overall, it is hoped that more effective strategies can be designed for the development of iron chelating and or other antioxidant drugs to be used in medicine based on approaches similar to the development of orphan drugs and also of other drugs originating from academic initiatives, such as, for example, the cases of L1 and ferric maltol [336,337]. Within this context, the formation of an association or a consortium of groups of expert academics related to all aspects of drug development, including chemistry, pharmacology, toxicology, clinical trial design, monitoring, and evaluation (phases I–IV), as well as expert groups on regulatory drug affairs, could increase the overall prospects for antioxidant drug development and clinical use in different diseases [50]. Similar groups have been previously formed to oversee developments with the orphan drug L1 [339,340].

## 6. Conclusions

A plethora of scientific and clinical evidence suggests that there is an urgent need for the introduction of antioxidant drugs for clinical use in different conditions involving FR pathology, including cancer, neurodegenerative, cardiovascular, kidney, liver, and many other diseases. Despite the lack of interest in antioxidant drug development by pharmaceutical companies, millions of people are taking antioxidant nutraceuticals daily and attending alternative medicine clinics for protection against diseases caused by FR toxicity, heavy metal toxicity, nutrient deficiency, etc. Similarly, many efforts have been undertaken by the academic community for the development of antioxidant drugs in different diseases, with some successes so far, for example, in the repurposing of the iron chelating/antioxidant drug L1 and the glutathione precursor drug NAC.

New academic approaches and strategies are required for the development of antioxidant drugs, including efforts within the framework of orphan drug regulatory requirements, which is a less tedious and expensive procedure than formal drug development. In this context, new initiatives and efforts should be focused on the targeting and treatment or improvement of treatment of diseases, especially those with no proven effective therapies, such as many types of cancer, cancer metastasis, drug resistance, neurodegenerative, cardiovascular diseases, etc. This process could be facilitated through concerted efforts for the repurposing, for example, of the iron chelating/antioxidant drugs L1 and DF and their combination, as well as combinations with other drugs with antioxidant properties such as NAC. In each disease, significant therapeutic improvement, following randomised clinical trials, should be shown by using the proposed antioxidant(s) drugs or their combination with other drugs.

The antioxidant drug strategy should be specific for each target of oxidative toxicity damage or other related metabolic or cellular functional pathways in each disease, and should not only rely on the improvement of antioxidant biomarker parameters, but also on clinical improvement, which should be measurable by monitoring the pathological indices related to the disease. Furthermore, the antioxidant therapy should be shown to proceed with no serious toxic side effects, and also with reduced overall risk and increased benefit for the treated patients.

In many of the clinical studies using ascorbate, EDTA, and the iron chelating/antioxidant drugs L1 and DF, a wide variation in the doses and sometimes wrong posology, as well as a lack of ferrikinetic and pharmacodynamic data and other monitoring parameters, have been observed, which resulted in ambiguous or inconclusive findings. In this context, for each disease, full transparency in the design of the antioxidant drug therapeutic protocols, including effective posology and appropriate monitoring of pharmacodynamic and other parameters, is necessary. Furthermore, variable protocols and monitoring will be needed, which, for example, may be related to the prevention, treatment, or post-treatment effects of a disease, and also for long-term or short-term or intermittent antioxidant drug administration.

Despite the many limitations and failures in the clinical testing of a wide number of potential antioxidants, including iron chelating/antioxidant drugs, some encouraging results have been noted in some cases, especially in the use of the iron chelating/antioxidant drug L1 in the treatment of different non-iron-loaded clinical conditions. Further evaluation of antioxidant drug combination therapies, including the combination of L1 with NAC and also of other antioxidants, appears to increase the prospects of antioxidant drug therapies in different diseases. Further strategies should also be designed for identifying synergistic combination therapies of proposed antioxidant drug(s) with other drugs used for the treatment of the underlying disease.

New antioxidant strategies may also involve the identification of pro-drugs, such as aspirin, which is biotransformed and partly forms iron chelating/antioxidant metabolites with possible clinical application as antioxidant chemoprevention and also in therapeutic interventions. Future strategies may also involve the development of iron chelating/antioxidants for the modulation of ferroptosis, a programmed cell-death process identified in almost all diseases.

More concerted efforts are required, mostly by the academic community, to increase the prospects for the repurposing of iron chelating/antioxidant drugs and also the development of new iron chelating/antioxidants drugs for clinical use in many diseases of OST, and also of diseases associated with ferroptosis. In this context, the formation of a consortium of expert academics on regulatory drug affairs, and also of other expert groups related to different aspects of drug development, including clinical trial design, monitoring, and evaluation, could increase the overall prospects for the development of different antioxidant drugs for clinical use in different diseases.

## Figures and Tables

**Table 1:** General clinical and non-clinical properties of the iron chelating/antioxidant drugs deferoxamine, deferasirox, and deferiprone.

**CLINICAL IRON-RELATED EFFECTS OF THE IRON CHELATING DRUGS**
(a) Recommended doses in iron-loaded thalassaemia patients: DF subcutaneously or intravenously 40–60 mg/kg/day. Oral DFRA 20–40 mg/kg/day. Oral L1 75–100 mg/kg/day.
(b) Recommended doses of oral L1 in neurodegenerative and other non-iron-loaded patients: Minimum single dose of L1 25 mg/kg/day. Repeated administration 2–4 times per day.
(c) Iron-loaded patient compliance with iron chelating drugs: Lower compliance with subcutaneous or intravenous DF in comparison to oral L1 and DFRA.
(d) Non-iron-loaded patient compliance and toxicity: High compliance with L1. DF and DFRA are generally toxic in this patient category, especially with prolonged treatments.
(e) Differential iron removal from various organs of iron-loaded patients: Efficacy is related to dose for all chelators. L1 causes preferential iron removal from the heart and DFRA from the liver. DF causes preferential iron removal mainly from the liver and less from the heart.
(f) Increase in excretion of metal ions other than iron: DF and L1 cause an increase in aluminium excretion in renal dialysis patients. DFRA causes increases in aluminium and other toxic metal absorption.
(g) Iron mobilisation and excretion of chelator metabolite–iron complexes: Several DF metabolites have iron chelation potential and cause an increase in iron excretion, but not the L1 glucuronide or the DFRA glucuronide metabolites.
(h) Combination chelation therapy: L1, DF, and DFRA combinations are more effective in iron excretion than monotherapy. The ICOC L1 and DF combination protocol has been shown to cause normalisation of the iron stores in iron-loaded thalassaemia patients.
(i) Synergism with reducing agents: Ascorbate act synergistically with DF but not L1 or DFRA for increasing iron excretion.
(j) Route of elimination of chelator and its iron complex: DF: Urine and faeces. L1: urine. DFRA: Almost exclusively in faeces and less than 0.1–8% in urine.
(k) Enterohepatic circulation: Limited small amounts of DFRA and metabolites, but not DF and L1.
**PHARMACOKINETIC, METABOLIC, AND ANTIOXIDANT PROPERTIES OF THE CHELATING DRUGS**
(a) Metabolite(s): DF: A number of metabolites, cleared mainly through the urine, some with iron chelating properties. L1: Glucuronide conjugate, cleared through the urine but has no iron chelation properties. DFRA: Glucuronide conjugates are cleared through the faeces and have no iron chelation properties.
(b) T1/2 absorption: DFRA: 1–2 h. L1: 0.7–32 min.
(c) T max of the chelator: DFRA: Mostly 4–6 h. L1: Mostly within 1 h.
(d) T max of the metabolite L1-glucuronide: 1–3 h.
(e) T max of the iron complex: DFRA: 1–6 h at 20 mg/kg and 4–8 h at 40 mg/kg. L1: Estimated within 1 h.
(f) T1/2 elimination of chelator: DF: 5–10 min. DFRA: 19 ± 6.5 h at 20 and 40 mg/kg. L1: 47–134 min at 35–71 mg/kg.
(g) T1/2 elimination of the iron complex: DF: 90 min. DFRA: 17.2 ± 7.8 h at 20 mg/kg and 17.7 ± 5.1 h at 40 mg/kg. L1: Estimated within 47–134 min.
(h) Antioxidant potential at recommended doses in iron-loaded patients: All three chelating drugs are effective.
(i) Antioxidant potential at effective doses in non-iron-loaded patients: L1 > DF > DFRA.
(j) Crossing of the blood-brain barrier and targeting diseases of the brain in non-iron-loaded patients: Oral L1, intranasal DF, but not DFRA.
**CHEMICAL AND PHYSICOCHEMICAL PROPERTIES OF THE CHELATING DRUGS**
(a) Molecular weight of chelators: DF: 561. DFRA: 373. L1: 139.
(b) Molecular weight of chelator–iron complexes: DF: 617. DFRA: 798. L1: 470.
(c) Stoichiometry of chelator–iron complexes at pH 7.4: 1DF:1Fe. 2DFRA:1Fe. 3L1:1Fe.
(d) Charge of chelators at pH 7.4: DF positive. DFRA negative. L1 neutral.
(e) Charge of iron complexes at pH 7.4: DF positive. DFRA negative. L1 neutral.
(f) Partition coefficient of chelators (n-octanol/water): DF: 0.02. DFRA: 6.3. L1: 0.19.
(g) Partition coefficient of chelator–iron complexes (n-octanol/water): DF: 0.02. L1: 0.05.
(h) Stability constant (Log β) of chelator–iron complexes: DF: 31. DFRA: 27. L1: 35.

Abbreviations: DF: deferoxamine. L1: deferiprone. DFRA: deferasirox. ICOC: International committee on chelation. T max: time of maximum concentration in blood. T1/2: half-life in blood. For further information see references [10,33,50,90,92].

**Table 2:** The unique clinical and other properties of deferiprone and prospects for its repurposing as a universal iron chelating/antioxidant in free radical pathology and ferroptosis.

**DRUG PROPERTIES, USES, AND AVAILABILITY OF DEFERIPRONE**
(a) Iron chelating drug in the essential WHO list of medicines, used worldwide. Regulatory-approved drug by EMA and FDA.
(b) One step, simple chemical synthesis, inexpensive, and wide availability worldwide.
(c) White crystalline solid, stable at room temperature or refrigerator (4–5 °C) for more than 20 years. Main formulations available are tablets or capsules.
(d) Orally effective, rapid absorption, appearance in blood, and wide organ distribution in minutes. Iron-binding and antioxidant effects throughout the body.
(e) Good compliance in iron-loaded and non-iron-loaded patients.
(f) Ability to cross the blood-brain barrier and remove excess iron from the brain and treat malignant, neurological, and microbial diseases affecting the brain.
(g) The only drug causing iron removal from transferrin in iron-loaded patients.
(h) Iron donation to apo-transferrin in normal volunteers and non-iron-loaded patients.
(i) Potent antioxidant activity through inhibition of iron and copper catalytic production of free radicals in many in vitro, in vivo, and clinical models.
(j) Inhibition of ferroptosis and cuproptosis involved in many diseases of free radical pathology.
(k) Use in metal intoxication diseases, including those related to iron, copper, aluminium, zinc, gallium, indium, uranium, and plutonium.
(l) Drug combination therapies with DF, DFRA, EDTA, DTPA, and other natural or synthetic chelators.
**SAFETY AND CLINICAL EFFECTS OF DEFERIPRONE IN IRON-LOADED THALASSAEMIA PATIENTS**
(a) Daily use in iron-loaded patients at high doses (75–100 mg/kg) for up to 30 years with no serious toxicity.
(b) Iron removal from all organs, especially the heart, which is the target organ of iron toxicity in transfused TM iron-loaded patients. Complete removal of excess iron from all iron storage organs when used as monotherapy or combination therapy with DF.
(c) Maintenance of normal iron stores in ex-iron-loaded transfused TM patients. Efficient iron removal from the heart and reduction of congestive cardiac failure in different iron-loaded diseases.
(d) Improvement of LVEF and endothelial cell function in TM and other conditions.
(e) Improvements in the antioxidant status, including increases in glutathione levels and in cellular function.
(f) Caused a decrease in the mortality rate in transfused TM patients and the transition of thalassaemia from a fatal to a chronic disease.
**CLINICAL EFFECTS AND POSOLOGY OF DEFERIPRONE IN NON-IRON-LOADED PATIENT CATEGORIES**
(a) Removal of excess iron and aluminium in renal dialysis patients. Posology: 30–60 mg/kg/day.
(b) Increase in haemoglobin and improvement of anaemia in rheumatoid arthritis patients. Posology: Up to 50 mg/kg/day for a month.
(c) Fast resolution of fever and coma and rapid parasitaemia clearance in malaria patients. Posology: 100 mg/kg/day for a week.
(d) Antiretroviral action and release of innate apoptotic defence of HIV-infected cells from viral blockade in HIV patients. Posology: 3 × 33 mg/kg/day and 3 × 50 mg/kg/day for 35 days were used.
(e) Removal of excess iron from the brain and cardiac iron with improvement of cardiac function in aceruloplasminemia. Posology: 15–75 mg/kg/day for up to 6 months.
(f) Removal of excess iron from the brain and improvement in motor scores in Parkinson’s disease patients. Posology: 15–30 mg/kg/day for up to a year.
(g) Removal of excess iron from the brain in Alzheimer’s disease patients. Posology: 15–30 mg/kg/day for up to a year.
(h) Removal of excess iron from the brain and reduction in neuropathy and ataxic gait in Friedreich’s Ataxia patients. Posology: 20–30 mg/kg/day for six months.
(i) Removal of excess iron from the brain and slowing of disease progression in neurodegeneration with brain iron accumulation (NBIA) patients. Posology: 15–50 mg/kg/day for six or more months.
(j) Removal of excess iron from the brain and stability of the overall clinical neurological picture in pantothenate kinase 2-associated neurodegeneration (PKAN) patients. Posology: 15–30 mg/kg/day for up to four years.
(k) Significant reduction in urinary protein, no significant changes in serum creatinine in glomerulonephritis patients. Persistent drop in mean albumin/creatinine ratio, 9-month stable renal function in diabetic nephropathy patients. Posology: 50 mg/kg/day for 6–9 months.
(l) Eradication of cancer stem cells through selective targeting of mitochondria in breast cancer patients.
(m) Inhibition of prostate cancer proliferation in prostate cancer patients.

Abbreviations: DF: deferoxamine. DFRA: deferasirox. DTPA: diethylenetriaminepentaacetic acid. EDTA: ethylenediaminetetraacetic acid. L1: deferiprone. LVEF: left-ventricular ejection fraction. NBIA: Neurodegeneration with brain iron accumulation. PKAN: pantothenate kinase 2-associated neurodegeneration. TM: thalassaemia major. For further information see references [33,50,90,92,114,115,116,117,118,119,121].

## Abbreviations

ADMET	absorption, distribution, metabolism, elimination, and toxicity
DF	deferoxamine
DFRA	deferasirox
DTPA	diethylenetriaminepenntaacetic acid
EDTA	ethylenediaminetetraacetic acid
FR	free radicals
GSH	reduced glutathione
IV	intravenous
L1	deferiprone
L1-G	deferiprone glucuronide metabolite
LVEF	left- venricular ejection fraction
MRI	magnetic resonance imaging
NBIA	neurodegeneration with brain iron accumulation
NCA	N-acetylcysteine
OST	oxidative stress toxicity
PKAN	pantothenate kinase 2-associated neurodegeneration
ROS	reactive oxygen species
TM	thalassaemia major
TAOC	total antioxidant capacity
SC	subcutaneous

## References

[1] Denisov E.T., Afanas’ev I.B. (2005). Oxidation and Antioxidants in Organic Chemistry and Biology.

[2] Halliwell B., Gutteridge J.M.C., Cross C.E. (1992). Free radicals, antioxidants and human disease: Where are we now?. J. Lab. Clin. Med..

[3] Reddy V.P. (2023). Oxidative Stress in Health and Disease. Biomedicines.

[4] Iakovou E., Kourti M. (2022). A Comprehensive Overview of the Complex Role of Oxidative Stress in Aging, The Contributing Environmental Stressors and Emerging Antioxidant Therapeutic Interventions. Front. Aging Neurosci..

[5] Stangherlin A., Reddy A.B. (2013). Regulation of circadian clocks by redox homeostasis. J. Biol. Chem..

[6] Forman H.J. (2016). Redox signaling: An evolution from free radicals to aging. Free Radic. Biol. Med..

[7] Su Z., Hu Q., Li X., Wang Z., Xie Y. (2024). The Influence of Circadian Rhythms on DNA Damage Repair in Skin Photoaging. Int. J. Mol. Sci..

[8] Arevalo J.A., Vázquez-Medina J.P. (2018). The Role of Peroxiredoxin 6 in Cell Signaling. Antioxidants.

[9] Chakraborty P., Dewanjee S. (2024). Unrevealing the mechanisms behind the cardioprotective effect of wheat polyphenolics. Arch. Toxicol..

[10] Kontoghiorghe C.N., Kolnagou A., Kontoghiorghes G.J. (2015). Phytochelators Intended for Clinical Use in Iron Overload, Other Diseases of Iron Imbalance and Free Radical Pathology. Molecules.

[11] Black H.S., Boehm F., Edge R., Truscott T.G. (2020). The Benefits and Risks of Certain Dietary Carotenoids that Exhibit both Anti- and Pro-Oxidative Mechanisms—A Comprehensive Review. Antioxidants.

[12] Pagano G., Talamanca A.A., Castello G., Cordero M.D., D’iSchia M., Gadaleta M.N., Pallardó F.V., Petrović S., Tiano L., Zatterale A. (2014). Oxidative stress and mitochondrial dysfunction across broad-ranging pathologies: Toward mitochondria-targeted clinical strategies. Oxidative Med. Cell. Longev..

[13] Vyas S., Zaganjor E., Haigis M.C. (2016). Mitochondria and Cancer. Cell.

[14] Kathiresan D.S., Balasubramani R., Marudhachalam K., Jaiswal P., Ramesh N., Sureshbabu S.G., Puthamohan V.M., Vijayan M. (2025). Role of Mitochondrial Dysfunctions in Neurodegenerative Disorders: Advances in Mitochondrial Biology. Mol. Neurobiol..

[15] Chandimali N., Bak S.G., Park E.H., Lim H.-J., Won Y.-S., Kim E.-K., Park S.-I., Lee S.J. (2025). Free radicals and their impact on health and antioxidant defenses: A review. Cell Death Discov..

[16] D’Orazio J., Jarrett S., Amaro-Ortiz A., Scott T. (2013). UV radiation and the skin. Int. J. Mol. Sci..

[17] Black H.S. (2004). Reassessment of a free radical theory of cancer with emphasis on ultraviolet carcinogenesis. Integr. Cancer Ther..

[18] Flora S.J., Shrivastava R., Mittal M. (2013). Chemistry and pharmacological properties of some natural and synthetic antioxidants for heavy metal toxicity. Curr. Med. Chem..

[19] Xiao C., Lai D. (2025). Impact of oxidative stress induced by heavy metals on ovarian function. J. Appl. Toxicol..

[20] Pan Z., Gong T., Liang P. (2024). Heavy Metal Exposure and Cardiovascular Disease. Circ. Res..

[21] Marant-Micallef C., Shield K.D., Vignat J., Cléro E., Kesminiene A., Hill C., Rogel A., Vacquier B., Bray F., Laurier D. (2019). The risk of cancer attributable to diagnostic medical radiation: Estimation for France in 2015. Int. J. Cancer.

[22] Ambra R., Lucchetti S., Pastore G. (2022). A Review of the Effects of Olive Oil-Cooking on Phenolic Compounds. Molecules.

[23] Gutteridge J.M. (1995). Lipid peroxidation and antioxidants as biomarkers of tissue damage. Clin. Chem..

[24] Evans M.D., Dizdaroglu M., Cooke M.S. (2004). Oxidative DNA damage and disease: Induction, repair and significance. Mutat. Res..

[25] Stadtman E.R., Levine R.L. (2003). Free radical-mediated oxidation of free amino acids and amino acid residues in proteins. Amino Acids.

[26] Chen X., Sun-Waterhouse D., Yao W., Li X., Zhao M., You L. (2021). Free radical-mediated degradation of polysaccharides: Mechanism of free radical formation and degradation, influence factors and product properties. Food Chem..

[27] Kern C., Bonventre J.V., Justin A.W., Kashani K., Reynolds E., Siew K., Davis B., Karakoy H., Grzesiak N., Bailey D.M. (2025). Necrosis as a fundamental driver of loss of resilience and biological decline: What if we could intervene?. Oncogene.

[28] Pompella A., Corti A. (2015). Editorial: The changing faces of glutathione, a cellular protagonist. Front. Pharmacol..

[29] Umeno A., Biju V., Yoshida Y. (2017). In vivo ROS production and use of oxidative stress-derived biomarkers to detect the onset of diseases such as Alzheimer’s disease, Parkinson’s disease, and diabetes. Free Radic. Res..

[30] Topic A., Francuski D., Markovic B., Stankovic M., Dobrivojevic S., Drca S., Radojkovic D. (2013). Gender-related reference intervals of urinary 8-oxo-7,8-dihydro-2′-deoxyguanosine determined by liquid chromatography-tandem mass spectrometry in Serbian population. Clin. Biochem..

[31] Didier A.J., Stiene J., Fang L., Watkins D., Dworkin L.D., Creeden J.F. (2023). Antioxidant and Anti-Tumor Effects of Dietary Vitamins A, C, and E. Antioxidants.

[32] Galaris D., Barbouti A., Pantopoulos K. (2019). Iron homeostasis and oxidative stress: An intimate relationship. Biochim. Biophys. Acta Mol. Cell Res..

[33] Kontoghiorghes G.J., Kontoghiorghe C.N. (2020). Iron and Chelation in Biochemistry and Medicine: New Approaches to Controlling Iron Metabolism and Treating Related Diseases. Cells.

[34] Pantopoulos K., Porwal S.K., Tartakoff A., Devireddy L. (2012). Mechanisms of mammalian iron homeostasis. Biochemistry.

[35] Dixon S.J., Lemberg K.M., Lamprecht M.R., Skouta R., Zaitsev E.M., Gleason C.E., Patel D.N., Bauer A.J., Cantley A.M., Yang W.S. (2012). Ferroptosis: An iron-dependent form of nonapoptotic cell death. Cell.

[36] Xie Y., Hou W., Song X., Yu Y., Huang J., Sun X., Kang R., Tang D. (2016). Ferroptosis: Process and function. Cell Death Differ..

[37] Cao J.Y., Dixon S.J. (2016). Mechanisms of ferroptosis. Cell. Mol. Life Sci..

[38] Liu L., Li L., Li M., Luo Z. (2021). Autophagy-Dependent Ferroptosis as a Therapeutic Target in Cancer. ChemMedChem.

[39] Mou Y., Wang J., Wu J., He D., Zhang C., Duan C., Li B. (2019). Ferroptosis, a new form of cell death: Opportunities and challenges in cancer. J. Hematol. Oncol..

[40] Catapano A., Cimmino F., Petrella L., Pizzella A., D’ANgelo M., Ambrosio K., Marino F., Sabbatini A., Petrelli M., Paolini B. (2025). Iron metabolism and ferroptosis in health and diseases: The crucial role of mitochondria in metabolically active tissues. J. Nutr. Biochem..

[41] Teschke R., Eickhoff A. (2024). Wilson Disease: Copper-Mediated Cuproptosis, Iron-Related Ferroptosis, and Clinical Highlights, with Comprehensive and Critical Analysis Update. Int. J. Mol. Sci..

[42] Mao C., Wang M., Zhuang L., Gan B. (2024). Metabolic cell death in cancer: Ferroptosis, cuproptosis, disulfidptosis, and beyond. Protein Cell.

[43] Lu K., Wijaya C.S., Yao Q., Jin H., Feng L. (2025). Cuproplasia and cuproptosis, two sides of the coin. Cancer Commun..

[44] Kontoghiorghe C.N., Kolnagou A., Kontoghiorghes G.J. (2013). Potential clinical applications of chelating drugs in diseases targeting transferrin-bound iron and other metals. Expert Opin. Investig. Drugs.

[45] Arosio P. (2022). New Advances in Iron Metabolism, Ferritin and Hepcidin Research. Int. J. Mol. Sci..

[46] Kontoghiorghes G.J. (2024). The Importance and Essentiality of Natural and Synthetic Chelators in Medicine: Increased Prospects for the Effective Treatment of Iron Overload and Iron Deficiency. Int. J. Mol. Sci..

[47] Sheppard L.N., Kontoghiorghes G.J. (1993). Competition between deferiprone, desferrioxamine and other chelators for iron and the effect of other metals. Arzneimittelforschung.

[48] Carlsen M.H., Halvorsen B.L., Holte K., Bøhn S.K., Dragland S., Sampson L., Willey C., Senoo H., Umezono Y., Sanada C. (2010). The total antioxidant content of more than 3100 foods, beverages, spices, herbs and supplements used worldwide. Nutr. J..

[49] Puri V., Nagpal M., Singh I., Singh M., Dhingra G.A., Huanbutta K., Dheer D., Sharma A., Sangnim T. (2022). A Comprehensive Review on Nutraceuticals: Therapy Support and Formulation Challenges. Nutrients.

[50] Kontoghiorghe C.N., Andreou N., Constantinou K., Kontoghiorghes G.J. (2014). World health dilemmas: Orphan and rare diseases, orphan drugs and orphan patients. World J. Methodol..

[51] Timoshnikov V.A., Selyutina O.Y., Polyakov N.E., Didichenko V., Kontoghiorghes G.J. (2022). Mechanistic Insights of Chelator Complexes with Essential Transition Metals: Antioxidant/Pro-Oxidant Activity and Applications in Medicine. Int. J. Mol. Sci..

[52] Kontoghiorghes G.J., Kontoghiorghe C.N. (2019). Prospects for the introduction of targeted antioxidant drugs for the prevention and treatment of diseases related to free radical pathology. Expert Opin. Investig. Drugs.

[53] Bruzzese A., Martino E.A., Mendicino F., Lucia E., Olivito V., Bova C., Filippelli G., Capodanno I., Neri A., Morabito F. (2023). Iron chelation therapy. Eur. J. Haematol..

[54] Wang L.E., Muttar S., Badawy S.M. (2025). The challenges of iron chelation therapy in thalassemia: How do we overcome them?. Expert Rev. Hematol..

[55] Inusa B.P., Atoyebi W., Andemariam B., Hourani J.N., Omert L. (2023). Global burden of transfusion in sickle cell disease. Transfus. Apher. Sci..

[56] Navaneethabalakrishnan S., An X., Vinchi F. (2024). Heme- and iron-activated macrophages in sickle cell disease: An updated perspective. Curr. Opin. Hematol..

[57] Küpesiz F.T., Hazar V., Eker N., Guler E., Yesilipek M.A., Tuysuz G., Kupesiz A. (2020). Retrospective Evaluation of Relationship Between Iron Overload and Transplantation Complications in Pediatric Patient Who Underwent Allogeneic Stem Cell Transplantation Due to Acute Leukemia and Myelodysplastic Syndrome. J. Pediatr. Hematol. Oncol..

[58] Baskin-Miller J., Carson S., Jaffray J., Fletcher C., Singer J., Freyer D.R., Wood J., Coates T.D., Denton C.C. (2024). Transfusional hemosiderosis in childhood cancer patients and survivors. Pediatr. Blood Cancer.

[59] Gao Q., Zhou Y., Chen Y., Hu W., Jin W., Zhou C., Yuan H., Li J., Lin Z., Lin W. (2025). Role of iron in brain development, aging, and neurodegenerative diseases. Ann. Med..

[60] Klein H.G., Spahn D.R., Carson J.L. (2007). Red blood cell transfusion in clinical practice. Lancet.

[61] Cazzola M., Borgna-Pignatti C., Locatelli F., Ponchio L., Beguin Y., De Stefano P. (1997). A moderate transfusion regimen may reduce iron loading in beta-thalassemia major without producing excessive expansion of erythropoiesis. Transfusion.

[62] Iancu T.C. (1983). Iron overload. Mol. Asp. Med..

[63] Pippard M.J., Weatherall D.J. (1984). Iron absorption in non-transfused iron loading anaemias: Prediction of risk for iron loading, and response to iron chelation treatment, in beta thalassaemia intermedia and congenital sideroblastic anaemias. Haematologia.

[64] Zurlo M., De Stefano P., Borgna-Pignatti C., Di Palma A., Melevendi C., Piga A., Di Gregorio F., Burattini M., Terzoli S. (1989). Survival and causes of death in thalassaemia major. Lancet.

[65] Modell B., Khan M., Darlison M. (2000). Survival in beta-thalassaemia major in the UK: Data from the UK Thalassaemia Register. Lancet.

[66] Telfer P., Coen P.G., Christou S., Hadjigavriel M., Kolnakou A., Pangalou E., Pavlides N., Psiloines M., Simamonian K., Skordos G. (2006). Survival of medically treated thalassemia patients in Cyprus. Trends and risk factors over the period 1980–2004. Haematologica.

[67] Kolnagou A., Kontoghiorghes G.J. (2009). Advances in the prevention and treatment are changing thalassemia from a fatal to a chronic disease. experience from a Cyprus model and its use as a paradigm for future applications. Hemoglobin.

[68] Modell B., Khan M., Darlison M., Westwood M.A., Ingram D., Pennell D.J. (2008). Improved survival of thalassaemia major in the UK and relation to T2* cardiovascular magnetic resonance. J. Cardiovasc. Magn. Reson..

[69] Au W.Y., Lee V., Lau C.W., Yau J., Chan D., Chan E.Y.T., Cheung W.W.W., Ha S.Y., Kho B., Lee C.Y. (2011). A synopsis of current care of thalassaemia major patients in Hong Kong. Hong Kong Med. J..

[70] Maggio A., Filosa A., Vitrano A., Aloj G., Kattamis A., Ceci A., Fucharoen S., Cianciulli P., Grady R.W., Prossomariti L. (2011). Iron chelation therapy in thalassemia major: A systematic review with meta-analyses of 1520 patients included on randomized clinical trials. Blood Cells Mol. Dis..

[71] Kontoghiorghes G.J. (2023). Iron Load Toxicity in Medicine: From Molecular and Cellular Aspects to Clinical Implications. Int. J. Mol. Sci..

[72] Adams P.C. (2015). Epidemiology and diagnostic testing for hemochromatosis and iron overload. Int. J. Lab. Hematol..

[73] Brissot P., Troadec M.-B., Loréal O., Brissot E. (2019). Pathophysiology and classification of iron overload diseases; update 2018. Transfus. Clin. Biol..

[74] Gao J., Zhou Q., Wu D., Chen L. (2021). Mitochondrial iron metabolism and its role in diseases. Clin. Chim. Acta.

[75] Shah S.V., Rajapurkar M.M. (2009). The Role of Labile Iron in Kidney Disease and Treatment with Chelation. Hemoglobin.

[76] Kyriacou K., Michaelides Y., Senkus R., Simamonian K., Pavlides N., Antoniades L., Zambartas C. (2000). Ultrastructural pathology of the heart in patients with beta-thalassaemia major. Ultrastruct. Pathol..

[77] Vassiliou E., Farias-Pereira R. (2023). Impact of Lipid Metabolism on Macrophage Polarization: Implications for Inflammation and Tumor Immunity. Int. J. Mol. Sci..

[78] Denz H., Huber P., Landmann R., Orth B., Wachter H., Fuchs D. (1992). Association between the activation of macrophages, changes of iron metabolism and the degree of anaemia in patients with malignant disorders. Eur. J. Haematol..

[79] Waldvogel D., van Gelderen P., Hallett M. (1999). Increased iron in the dentate nucleus of patients with Friedrich’s ataxia. Ann. Neurol..

[80] Economides C.P., Soteriades E.S., Hadjigavriel M., Seimenis I., Karantanas A. (2013). Iron deposits in the knee joints of a thalassemic patient. Acta Radiol. Short Rep..

[81] Meloni A., Pistoia L., Gamberini M.R., Ricchi P., Cecinati V., Sorrentino F., Cuccia L., Allò M., Righi R., Fina P. (2021). The Link of Pancreatic Iron with Glucose Metabolism and Cardiac Iron in Thalassemia Intermedia: A Large, Multicenter Observational Study. J. Clin. Med..

[82] Lehéricy S., Roze E., Goizet C., Mochel F. (2020). MRI of neurodegeneration with brain iron accumulation. Curr. Opin. Neurol..

[83] Drayer B.P., Olanow W., Burger P., Johnson G.A., Herfkens R., Riederer S. (1986). Parkinson plus syndrome: Diagnosis using high field MR imaging of brain iron. Radiology.

[84] Norfray J.F., Chiaradonna N.L., Heiser W.J., Song S.H., Manyam B.V., Devleschoward A.B., Eastwood L.M. (1988). Brain iron in patients with Parkinson disease: MR visualization using gradient modification. Am. J. Neuroradiol..

[85] Bartzokis G., Sultzer D., Mintz J., Holt L.E., Marx P., Phelan C.K., Marder S.R. (1994). In vivo evaluation of brain iron in Alzheimer’s disease and normal subjects using MRI. Biol. Psychiatry.

[86] Brar S., Henderson D., Schenck J., Zimmerman E.A. (2009). Iron Accumulation in the Substantia Nigra of Patients with Alzheimer Disease and Parkinsonism. Arch. Neurol..

[87] Kobayashi M., Suhara T., Baba Y., Kawasaki N.K., Higa J.K., Matsui T. (2018). Pathological Roles of Iron in Cardiovascular Disease. Curr. Drug Targets.

[88] Nielsen J.B. (1963). Influence of desferrioxamine on the renal excretion of iron. Preliminary report. Acta Med. Scand..

[89] Wöhler F. (1963). The Treatment of Haemochromatosis with Desferrioxamine. Acta Haematol..

[90] Kontoghiorghes G.J., Kleanthous M., Kontoghiorghe C.N. (2020). The History of Deferiprone (L1) and the Paradigm of the Complete Treatment of Iron Overload in Thalassaemia. Mediterr. J. Hematol. Infect. Dis..

[91] Nisbet-Brown E., Olivieri N.F., Giardina P.J., Grady R.W., Neufeld E.J., Séchaud R., Krebs-Brown A.J., Anderson J.R., Alberti D., Sizer K.C. (2003). Effectiveness and safety of ICL670 in iron-loaded patients with thalassaemia: A randomised, double-blind, placebo-controlled, dose-escalation trial. Lancet.

[92] Kontoghiorghes G.J., Kontoghiorghe C.N. (2016). Efficacy and safety of iron-chelation therapy with deferoxamine, deferiprone, and deferasirox for the treatment of iron-loaded patients with non-transfusion-dependent thalassemia syndromes. Drug Des. Dev. Ther..

[93] Dézsi L., Vécsei L. (2014). Clinical implications of irregular ADMET properties with levodopa and other antiparkinson’s drugs. Expert Opin. Drug Metab. Toxicol..

[94] US Food and Drug Administration (2005). Clinical Review: Exjade (Deferasirox, ICL-670). https://www.accessdata.fda.gov/drugsatfda_docs/nda/2005/021882_s000_Exjade_BioPharmr.pdf.

[95] *Exjade (Deferasirox) Tablets for Oral Suspension [Prescribing Information]*; Novartis Pharmaceutical Corporation: East Hanover, NJ, USA, 2011. http://www.accessdata.fda.gov/drugsatfda_docs/label/2010/021882s010lbl.pdf.

[96] Kontoghiorghes G.J., Kolnagou A., Peng C.T., Shah S.V., Aessopos A. (2010). Safety issues of iron chelation therapy in patients with normal range iron stores including thalassaemia, neurodegenerative, renal and infectious diseases. Expert Opin. Drug Saf..

[97] Kontoghiorghes G.J. (2013). A record number of fatalities in many categories of patients treated with deferasirox: Loopholes in regulatory and marketing procedures undermine patient safety and misguide public funds?. Expert Opin. Drug Saf..

[98] Chuang G.T., Tsai I.J., Tsau Y.K., Lu M.Y. (2015). Transfusion-dependent thalassaemic patients with renal Fanconi syndrome due to deferasirox use. Nephrology.

[99] Al-Khabori M., Bhandari S., Al-Huneini M., Al-Farsi K., Panjwani V., Daar S. (2013). Side effects of Deferasirox Iron Chelation in Patients with Beta Thalassemia Major or Intermedia. Oman Med. J..

[100] Naderi M., Sadeghi-Bojd S., Valeshabad A.K., Jahantigh A., Alizadeh S., Dorgalaleh A., Tabibian S., Bamedi T. (2013). A prospective study of tubular dysfunction in pediatric patients with Beta thalassemia major receiving deferasirox. Pediatr. Hematol. Oncol..

[101] Dee C.M., Cheuk D.K., Ha S.Y., Chiang A.K., Chan G.C. (2014). Incidence of deferasirox-associated renal tubular dysfunction in children and young adults with beta-thalassaemia. Br. J. Haematol..

[102] Maximova N., Gregori M., Simeone R., Sonzogni A., Zanon D., Boz G., D’Antiga L. (2018). Total body irradiation and iron chelation treatment are associated with pancreatic injury following pediatric hematopoietic stem cell transplantation. Oncotarget.

[103] Fucile C., Mattioli F., Marini V., Gregori M., Sonzogni A., Martelli A., Maximova N. (2018). What is known about deferasirox chelation therapy in pediatric HSCT recipients: Two case reports of metabolic acidosis. Ther. Clin. Risk Manag..

[104] Boelaert J.R., Fenves A.Z., Coburn J.W. (1991). Deferoxamine therapy and mucormycosis in dialysis patients: Report of an international registry. Am. J. Kidney Dis..

[105] Orton R.B., de Veber L.L., Sulh H.M. (1985). Ocular and auditory toxicity of long-term, high-dose subcutaneous deferoxamine therapy. Can. J. Ophthalmol..

[106] Cases A., Kelly J., Sabater F., Torras A., Griño M.C., Lopez-Pedret J., Revert L. (1990). Ocular and auditory toxicity in hemodialyzed patients receiving desferrioxamine. Nephron.

[107] Ioannides A.S., Panisello J.M. (2000). Acute respiratory distress syndrome in children with acute iron poisoning: The role of intravenous desferrioxamine. Eur. J. Pediatr..

[108] Cohen A.R., Galanello R., Piga A., De Sanctis V., Tricta F. (2003). Safety and effectiveness of long-term therapy with the oral iron chelator deferiprone. Blood.

[109] Ceci A., Baiardi P., Felisi M., Cappellini M.D., Carnelli V., De Sanctis V., Galanello R., Maggio A., Masera G., Piga A. (2002). The safety and effectiveness of deferiprone in a large-scale, 3-year study in Italian patients. Br. J. Haematol..

[110] Galanello R. (2007). Deferiprone in the treatment of transfusion-dependent thalassemia: A review and perspective. Ther. Clin. Risk Manag..

[111] Mazza P., Amurri B., Lazzari G., Masi C., Palazzo G., Spartera M.A., Giua R., Sebastio A.M., Suma V., De Marco S. (1998). Oral iron chelating therapy. A single center interim report on deferiprone (L1) in thalassemia. Haematologica.

[112] Calvaruso G., Vitrano A., Di Maggio R., Lai E., Colletta G., Quota A., Gerardi C., Rigoli L.C., Sacco M., Pitrolo L. (2015). Deferiprone versus deferoxamine in thalassemia intermedia: Results from a 5-year long-term Italian multicenter randomized clinical trial. Am. J. Hematol..

[113] Agarwal M.B. (1994). L1 arthropathy syndrome. J. Assoc. Physicians India.

[114] Kolnagou A., Kleanthous M., Kontoghiorghes G.J. (2022). Benefits and Risks in Polypathology and Polypharmacotherapy Challenges in the Era of the Transition of Thalassaemia from a Fatal to a Chronic or Curable Disease. Front. Biosci. (Elite Ed.).

[115] Kontoghiorghes G.J. (2023). The Vital Role Played by Deferiprone in the Transition of Thalassaemia from a Fatal to a Chronic Disease and Challenges in Its Repurposing for Use in Non-Iron-Loaded Diseases. Pharmaceuticals.

[116] Vreugdenhil G., Swaak A.J., Kontoghiorghes G.J., van Eijk H.G. (1989). Efficacy and safety of oral iron chelator L1 in anaemic rheumatoid arthritis patients. Lancet.

[117] van der Kraaij A.M., van Eijk H.G., Koster J.F. (1989). Prevention of postischemic cardiac injury by the orally active iron chelator 1,2-dimethyl-3-hydroxy-4-pyridone (L1) and the antioxidant (+)-cyanidanol-3. Circulation.

[118] Vreugdenhil G., Kontoghiorghes G.J., Van Eijk H.G., Swaak A.J. (1991). Impaired erythropoietin responsiveness to the anaemia in rheumatoid arthritis. A possible inverse relationship with iron stores and effects of the oral iron chelator 1,2-dimethyl-3-hydroxypyrid-4-one. Clin. Exp. Rheumatol..

[119] Kontoghiorghes G.J., Barr J., Baillod R.A. (1994). Studies of aluminium mobilization in renal dialysis patients using the oral chelator 1,2-dimethyl-3-hydroxypyrid-4-one. Arzneimittelforschung.

[120] Matthews A.J., Vercellotti G.M., Menchaca H.J., Bloch P.H., Michalek V.N., Marker P.H., Murar J., Buchwald H. (1997). Iron and atherosclerosis: Inhibition by the iron chelator deferiprone (L1). J. Surg. Res..

[121] Kontoghiorghes G.J., Neocleous K., Kolnagou A. (2003). Benefits and risks of deferiprone in iron overload in Thalassaemia and other conditions: Comparison of epidemiological and therapeutic aspects with deferoxamine. Drug Saf..

[122] Kontoghiorghes G.J., Barr J., Nortey P., Sheppard L. (1993). Selection of a new generation of orally active alpha-ketohydroxypyridine iron chelators intended for use in the treatment of iron overload. Am. J. Hematol..

[123] Olivieri N.F., Koren G., Matsui D., Liu P.P., Blendis L., Cameron R., McClelland R.A., Templeton D.M. (1992). Reduction of tissue iron stores and normalization of serum ferritin during treatment with the oral iron chelator L1 in thalassemia intermedia. Blood.

[124] Kolnagou A., Kleanthous M., Kontoghiorghes G.J. (2010). Reduction of body iron stores to normal range levels in thalassaemia by using a deferiprone/deferoxamine combination and their maintenance thereafter by deferiprone monotherapy. Eur. J. Haematol..

[125] Farmaki K., Tzoumari I., Pappa C., Chouliaras G., Berdoukas V. (2010). Normalisation of total body iron load with very intensive combined chelation reverses cardiac and endocrine complications of thalassaemia major. Br. J. Haematol..

[126] Kolnagou A., Kontoghiorghe C.N., Kontoghiorghes G.J. (2017). Prevention of Iron Overload and Long Term Maintenance of Normal Iron Stores in Thalassaemia Major Patients using Deferiprone or Deferiprone Deferoxamine Combination. Drug Res..

[127] Pennell D.J. (2005). T2* magnetic resonance and myocardial iron in thalassemia. Ann. N. Y. Acad. Sci..

[128] Papakonstantinou O., Alexopoulou E., Economopoulos N., Benekos O., Kattamis A., Kostaridou S., Ladis V., Efstathopoulos E., Gouliamos A., Kelekis N.L. (2009). Assessment of iron distribution between liver, spleen, pancreas, bone marrow, and myocardium by means of R2 relaxometry with MRI in patients with beta-thalassemia major. J. Magn. Reson. Imaging.

[129] Kolnagou A., Natsiopoulos K., Kleanthous M., Ioannou A., Kontoghiorghes G.J. (2013). Liver iron and serum ferritin levels are misleading for estimating cardiac, pancreatic, splenic and total body iron load in thalassemia patients: Factors influencing the heterogenic distribution of excess storage iron in organs as identified by MRI T2*. Toxicol. Mech. Methods.

[130] Iankova V., Karin I., Klopstock T., Schneider S.A. (2021). Emerging Disease-Modifying Therapies in Neurodegeneration with Brain Iron Accumulation (NBIA) Disorders. Front. Neurol..

[131] Wallis L.I., Paley M.N., Graham J.M., Grünewald R.A., Wignall E.L., Joy H.M., Griffiths P.D. (2008). MRI assessment of basal ganglia iron deposition in Parkinson’s disease. J. Magn. Reson. Imaging.

[132] Rajapurkar M.M., Lele S.S., Malavade T.S., Kansara M.R., Hegde U.N., Gohel K.D., Gang S.D., Shah S.V., Mukhopadhyay B.N. (2013). Serum catalytic Iron: A novel biomarker for coronary artery disease in patients on maintenance hemodialysis. Indian J. Nephrol..

[133] Wilson M.T., Reeder B.J. (2022). The peroxidatic activities of Myoglobin and Hemoglobin, their pathological consequences and possible medical interventions. Mol. Asp. Med..

[134] Li J.Y., Liu S.Q., Yao R.Q., Tian Y.P., Yao Y.M. (2021). A Novel Insight Into the Fate of Cardiomyocytes in Ischemia-Reperfusion Injury: From Iron Metabolism to Ferroptosis. Front. Cell Dev. Biol..

[135] Boddaert N., Le Quan Sang K.H., Rötig A., Leroy-Willig A., Gallet S., Brunelle F., Sidi D., Thalabard J.C., Munnich A., Cabantchik Z.I. (2007). Selective iron chelation in Friedreich ataxia: Biologic and clinical implications. Blood.

[136] Abbruzzese G., Cossu G., Balocco M., Marchese R., Murgia D., Melis M., Galanello R., Barella S., Matta G., Ruffinengo U. (2011). A pilot trial of deferiprone for neurodegeneration with brain iron accumulation. Haematologica.

[137] Cossu G., Abbruzzese G., Matta G., Murgia D., Melis M., Ricchi V., Galanello R., Barella S., Origa R., Balocco M. (2014). Efficacy and safety of deferiprone for the treatment of pantothenate kinase-associated neurodegeneration (PKAN) and neurodegeneration with brain iron accumulation (NBIA): Results from a four years follow-up. Park. Relat. Disord..

[138] Zorzi G., Zibordi F., Chiapparini L., Bertini E., Russo L., Piga A., Longo F., Garavaglia B., Aquino D., Savoiardo M. (2011). Iron-related MRI images in patients with pantothenate kinase-associated neurodegeneration (PKAN) treated with deferiprone: Results of a phase II pilot trial. Mov. Disord..

[139] Forni G.L., Balocco M., Cremonesi L., Abbruzzese G., Parodi R.C., Marchese R. (2008). Regression of symptoms after selective iron chelation therapy in a case of neurodegeneration with brain iron accumulation. Mov. Disord..

[140] Rohani M., Razmeh S., Shahidi G.A., Alizadeh E., Orooji M. (2018). A pilot trial of deferiprone in pantothenate kinase-associated neurodegeneration patients. Neurol. Int..

[141] Devos D., Cabantchik Z.I., Moreau C., Danel V., Mahoney-Sanchez L., Bouchaoui H., Gouel F., Rolland A.S., Duce J.A., Devedjian J.C. (2020). Conservative iron chelation for neurodegenerative diseases such as Parkinson’s disease and amyotrophic lateral sclerosis. J. Neural Transm..

[142] Devos D., Labreuche J., Rascol O., Corvol J.C., Duhamel A., Guyon Delannoy P., Poewe W., Compta Y., Pavese N., Růžička E. (2022). Trial of Deferiprone in Parkinson’s Disease. N. Engl. J. Med..

[143] Ayton S., Barton D., Brew B., Brodtmann A., Clarnette R., Desmond P., Devos D., Ellis K.A., Fazlollahi A., Fradette C. (2025). Deferiprone in Alzheimer Disease: A Randomized Clinical Trial. JAMA Neurol..

[144] Kontoghiorghes G.J. (2023). Drug Selection and Posology, Optimal Therapies and Risk/Benefit Assessment in Medicine: The Paradigm of Iron-Chelating Drugs. Int. J. Mol. Sci..

[145] Müller T., Möhr J.D. (2024). Negative findings from trials with NLY01 or deferiprone for Parkinson’s disease. Lancet Neurol..

[146] Aronson J.K., Heneghan C., Ferner R.E. (2023). Drug shortages. Part 1. Definitions and harms. Br. J. Clin. Pharmacol..

[147] Aronson J.K., Heneghan C., Ferner R.E. (2023). Drug shortages. Part 2: Trends, causes and solutions. Br. J. Clin. Pharmacol..

[148] Djordjevic D., McFadyen A., AAnderson J. (2023). Ethical challenges and opportunities in the development and approval of novel therapeutics for rare diseases. J. Med. Access.

[149] Rajapurkar M.M., Hegde U., Bhattacharya A., Alam M.G., Shah S.V. (2013). Effect of deferiprone, an oral iron chelator, in diabetic and non-diabetic glomerular disease. Toxicol. Mech. Methods.

[150] Saxena D., Spino M., Tricta F., Connelly J., Cracchiolo B.M., Hanauske A.R., D’Alliessi Gandolfi D., Mathews M.B., Karn J., Holland B. (2016). Drug-Based Lead Discovery: The Novel Ablative Antiretroviral Profile of Deferiprone in HIV-1-Infected Cells and in HIV-Infected Treatment-Naive Subjects of a Double-Blind, Placebo-Controlled, Randomized Exploratory Trial. PLoS ONE.

[151] Mohanty D., Ghosh K., Pathare A.V., Karnad D. (2002). Deferiprone (L1) as an adjuvant therapy for Plasmodium falciparum malaria. Indian J. Med. Res..

[152] Filosa A., Vitrano A., Rigano P., Calvaruso G., Barone R., Capra M., Cuccia L., Gagliardotto F., Pitrolo L., Prossomariti L. (2013). Long-term treatment with deferiprone enhances left ventricular ejection function when compared to deferoxamine in patients with thalassemia major. Blood Cells Mol. Dis..

[153] Pepe A., Meloni A., Capra M., Cianciulli P., Prossomariti L., Malaventura C., Putti M.C., Lippi A., Romeo M.A., Bisconte M.G. (2011). Deferasirox, deferiprone and desferrioxamine treatment in thalassemia major patients: Cardiac iron and function comparison determined by quantitative magnetic resonance imaging. Haematologica.

[154] Pepe A., Meloni A., Rossi G., Cuccia L., D’Ascola G.D., Santodirocco M., Cianciulli P., Caruso V., Romeo M.A., Filosa A. (2013). Cardiac and hepatic iron and ejection fraction in thalassemia major: Multicentre prospective comparison of combined deferiprone and deferoxamine therapy against deferiprone or deferoxamine monotherapy. J. Cardiovasc. Magn. Reson..

[155] Maggio A., Vitrano A., Lucania G., Capra M., Cuccia L., Gagliardotto F., Pitrolo L., Prossomariti L., Filosa A., Caruso V. (2012). Long-term use of deferiprone significantly enhances left-ventricular ejection function in thalassemia major patients. Am. J. Hematol..

[156] Morales N.P., Rodrat S., Piromkraipak P., Yamanont P., Paiboonsukwong K., Fucharoen S. (2022). Iron chelation therapy with deferiprone improves oxidative status and red blood cell quality and reduces redox-active iron in β-thalassemia/hemoglobin E patients. Biomed. Pharmacother..

[157] Tauchenová L., Křížová B., Kubánek M., Fraňková S., Melenovský V., Tintěra J., Kautznerová D., Malušková J., Jirsa M., Kautzner J. (2016). Successful Treatment of Iron-Overload Cardiomyopathy in Hereditary Hemochromatosis with Deferoxamine and Deferiprone. Can. J. Cardiol..

[158] Badat M., Kaya B., Telfer P. (2015). Combination-therapy with concurrent deferoxamine and deferiprone is effective in treating resistant cardiac iron-loading in aceruloplasminaemia. Br. J. Haematol..

[159] Elalfy M.S., Hamdy M., El-Beshlawy A., Ebeid F.S.E., Badr M., Kanter J., Inusa B., Adly A.A.M., Williams S., Kilinc Y. (2023). Deferiprone for transfusional iron overload in sickle cell disease and other anemias: Open-label study of up to 3 years. Blood Adv..

[160] Sudmantaitė V., Čelutkienė J., Glaveckaite S., Katkus R. (2020). Difficult diagnosis of cardiac haemochromatosis: A case report. Eur. Heart J. Case Rep..

[161] Tanner M.A., Galanello R., Dessi C., Smith G.C., Westwood M.A., Agus A., Roughton M., Assomull R., Nair S.V., Walker J.M. (2007). A randomized, placebo-controlled, double-blind trial of the effect of combined therapy with deferoxamine and deferiprone on myocardial iron in thalassemia major using cardiovascular magnetic resonance. Circulation.

[162] Chan S., Lian Q., Chen M.P., Jiang D., Ho J.T.K., Cheung Y.F., Chan G.C. (2018). Deferiprone inhibits iron overload-induced tissue factor bearing endothelial microparticle generation by inhibition oxidative stress induced mitochondrial injury, and apoptosis. Toxicol. Appl. Pharmacol..

[163] Eybl V., Caisová D., Koutenský J., Kontoghiorghes G.J. (1991). Influence of iron chelators, 1,2-dialkyl-3-hydroxypyridin-4-ones, on the lipid peroxidation and glutathione level in the liver of mice. Arch. Toxicol. Suppl..

[164] Sadrzadeh S.M., Nanji A.A., Price P.L. (1994). The oral iron chelator, 1,2-dimethyl-3-hydroxypyrid-4-one reduces hepatic-free iron, lipid peroxidation and fat accumulation in chronically ethanol-fed rats. J. Pharmacol. Exp. Ther..

[165] Kontoghiorghes G.J., Jackson M.J., Lunec J. (1986). In vitro screening of iron chelators using models of free radical damage. Free Radic. Res. Commun..

[166] Kontoghiorghe C.N., Kolnagou A., Kontoghiorghes G.J. (2014). Antioxidant targeting by deferiprone in diseases related to oxidative damage. Front. Biosci. (Elite Ed.).

[167] Kontoghiorghes G.J. (2009). Prospects for introducing deferiprone as potent pharmaceutical antioxidant. Front. Biosci. (Elite Ed.).

[168] Yuan Y., Yang X., Zhao Y., Flores J.J., Huang L., Gu L., Li R., Zhang X., Zhu S., Dong S. (2025). Mitochondrial ferritin upregulation by deferiprone reduced neuronal ferroptosis and improved neurological deficits via NDRG1/Yap pathway in a neonatal rat model of germinal matrix hemorrhage. J. Cereb. Blood Flow Metab..

[169] Mahoney-Sánchez L., Bouchaoui H., Ayton S., Devos D., Duce J.A., Devedjian J.C. (2021). Ferroptosis and its potential role in the physiopathology of Parkinson’s Disease. Prog. Neurobiol..

[170] Maheshwari S. (2023). Ferroptosis Signaling Pathways: Alzheimer’s Disease. Horm. Metab. Res..

[171] Costa I., Barbosa D.J., Benfeito S., Silva V., Chavarria D., Borges F., Remião F., Silva R. (2023). Molecular mechanisms of ferroptosis and their involvement in brain diseases. Pharmacol. Ther..

[172] Yang B., Yang K., Chen Y., Li Q., Chen J., Li S., Wu Y. (2025). Exposure of A2E to blue light promotes ferroptosis in the retinal pigment epithelium. Cell. Mol. Biol. Lett..

[173] Yamada K., Tazaki A., Ushio-Watanabe N., Usui Y., Takeda A., Matsunaga M., Suzumura A., Shimizu H., Zheng H., Ariefta N.R. (2023). Retinal ferroptosis as a critical mechanism for the induction of retinochoroiditis during ocular toxoplasmosis. Redox Biol..

[174] Ye Z., Yan Y., Jin F., Jiang J., Deng C., Wang L., Dong K. (2025). Deferiprone protects photoreceptors by inhibiting ferroptosis after experimental retinal detachment. Exp. Eye Res..

[175] Rayatpour A., Foolad F., Heibatollahi M., Khajeh K., Javan M. (2022). Ferroptosis inhibition by deferiprone, attenuates myelin damage and promotes neuroprotection in demyelinated optic nerve. Sci. Rep..

[176] Bao L., Zhao Y., Duan S., Wu K., Shan R., Liu Y., Yang Y., Chen Q., Song C., Li W. (2024). Ferroptosis is involved in Staphylococcus aureus-induced mastitis through autophagy activation by endoplasmic reticulum stress. Int. Immunopharmacol..

[177] Wang C., Xie L., Xing Y., Liu M., Yang J., Gao N., Cai Y. (2023). Iron-overload-induced ferroptosis in mouse cerebral toxoplasmosis promotes brain injury and could be inhibited by Deferiprone. PLoS Neglected Trop. Dis..

[178] Sharawy N., Aboulhoda B.E., Khalifa M.M., Morcos G.N., Morsy S.A.A.G., Alghamdi M.A., Khalifa I.M., Abd Algaleel W.A. (2024). Amelioration of nephrotoxicity by targeting ferroptosis: Role of NCOA4, IREB2, and SLC7a11 signaling. Braz. J. Med. Biol. Res..

[179] Linjacki S., Wang Y., Baath N., Mantle D., Yang G. (2024). H_2_S Protects from Rotenone-Induced Ferroptosis by Stabilizing Fe-S Clusters in Rat Cardiac Cells. Cells.

[180] El Hajj S., Canabady-Rochelle L., Fries-Raeth I., Gaucher C. (2024). A Smooth Muscle Cell-Based Ferroptosis Model to Evaluate Iron-Chelating Molecules for Cardiovascular Disease Treatment. Curr. Issues Mol. Biol..

[181] Tong J., Lan X.T., Zhang Z., Liu Y., Sun D.Y., Wang X.J., Ou-Yang S.X., Zhuang C.L., Shen F.M., Wang P. (2023). Ferroptosis inhibitor liproxstatin-1 alleviates metabolic dysfunction-associated fatty liver disease in mice: Potential involvement of PANoptosis. Acta Pharmacol. Sin..

[182] Peña-Montes D.J., Huerta-Cervantes M., Riveros-Rosas H., Manzo-Avalos S., Aguilera-Méndez A., Huerta M., Trujillo X., Cortés-Rojo C., Montoya-Pérez R., Salgado-Garciglia R. (2024). Iron chelation mitigates mitochondrial dysfunction and oxidative stress by enhancing nrf2-mediated antioxidant responses in the renal cortex of a murine model of type 2 diabetes. Mitochondrion.

[183] Seddiek H., Hanna M., Hamoud A.E.M., Elbaset M.A., Akabawy A.M.A., Kotb M.Z., Khalifa M.M. (2025). Deferiprone ameliorates cisplatin induced peripheral neurotoxicity via ferritinophagy adjustment. Sci. Rep..

[184] Lian G., Huang X.X., Zeng Y. (2023). Puerarin Induces Ferroptosis in Colorectal Cancer Cells via Triggering NCOA4 Upregulation. Nutr. Cancer.

[185] Yu W., Li Y., Gao C., Li D., Chen L., Dai B., Yang H., Han L., Deng Q., Bian X. (2024). MDH2 Promotes Hepatocellular Carcinoma Growth Through Ferroptosis Evasion via Stabilizing GPX4. Int. J. Mol. Sci..

[186] Park M., Cho Y.L., Choi Y., Min J.K., Park Y.J., Yoon S.J., Kim D.S., Son M.Y., Chung S.W., Lee H. (2023). Particulate matter induces ferroptosis by accumulating iron and dysregulating the antioxidant system. BMB Rep..

[187] Sun Y., Kinsela A.S., Waite T.D. (2022). Elucidation of alveolar macrophage cell response to coal dusts: Role of ferroptosis in pathogenesis of coal workers’ pneumoconiosis. Sci. Total Environ..

[188] Zhang P., Chen Y., Zhang S., Chen G. (2022). Mitochondria-Related Ferroptosis Drives Cognitive Deficits in Neonatal Mice Following Sevoflurane Administration. Front. Med..

[189] Nobuta H., Yang N., Ng Y.H., Marro S.G., Sabeur K., Chavali M., Stockley J.H., Killilea D.W., Walter P.B., Zhao C. (2019). Oligodendrocyte Death in Pelizaeus-Merzbacher Disease Is Rescued by Iron Chelation. Cell Stem Cell.

[190] Shen C., Yang Q., Chen K., Ma H., Wang X., Tong J., Shen Y., Cui H. (2024). Uncovering the role of ferroptosis in Bietti crystalline dystrophy and potential therapeutic strategies. Cell Commun. Signal..

[191] Paraskevaidis I.A., Iliodromitis E.K., Vlahakos D., Tsiapras D.P., Nikolaidis A., Marathias A., Michalis A., Kremastinos D.T. (2005). Deferoxamine infusion during coronary artery bypass grafting ameliorates lipid peroxidation and protects the myocardium against reperfusion injury: Immediate and long-term significance. Eur. Heart J..

[192] Gajardo Cortez A.I.J., Lillo-Moya J., San-Martín-Martinez D., Pozo-Martinez J., Morales P., Prieto J.C., Aguayo R., Puentes Á., Ramos C., Silva S. (2024). Safety and Pharmacokinetics of a Combined Antioxidant Therapy against Myocardial Reperfusion Injury: A Phase 1 Randomized Clinical Trial in Healthy Humans. Clin. Pharmacol. Drug Dev..

[193] Lamichhane A., Sharma S., Bastola B., Chhusyabaga B., Shrestha N., Poudel P. (2024). Unlocking the potential of deferoxamine: A systematic review on its efficacy and safety in alleviating myocardial ischemia-reperfusion injury in adult patients following cardiopulmonary bypass compared to standard care. Ther. Adv. Cardiovasc. Dis..

[194] Daglas M., Adlard P.A. (2018). The Involvement of Iron in Traumatic Brain Injury and Neurodegenerative Disease. Front. Neurosci..

[195] Selim M., Foster L.D., Moy C.S., Xi G., Hill M.D., Morgenstern L.B., Greenberg S.M., James M.L., Singh V., Clark W.M. (2019). Deferoxamine mesylate in patients with intracerebral haemorrhage (i-DEF): A multicentre, randomised, placebo-controlled, double-blind phase 2 trial. Lancet Neurol..

[196] Foster L., Robinson L., Yeatts S.D., Conwit R.A., Shehadah A., Lioutas V., Selim M., i-DEF Investigators (2022). Effect of Deferoxamine on Trajectory of Recovery After Intracerebral Hemorrhage: A Post Hoc Analysis of the i-DEF Trial. Stroke.

[197] Millán M., DeGregorio-Rocasolano N., Pérez de la Ossa N., Reverté S., Costa J., Giner P., Silva Y., Sobrino T., Rodríguez-Yáñez M., Nombela F. (2021). Targeting Pro-Oxidant Iron with Deferoxamine as a Treatment for Ischemic Stroke: Safety and Optimal Dose Selection in a Randomized Clinical Trial. Antioxidants.

[198] Schaefer B., Effenberger M., Zoller H. (2014). Iron metabolism in transplantation. Transpl. Int..

[199] Yamada N., Karasawa T., Wakiya T., Sadatomo A., Ito H., Kamata R., Watanabe S., Komada T., Kimura H., Sanada Y. (2020). Iron overload as a risk factor for hepatic ischemia-reperfusion injury in liver transplantation: Potential role of ferroptosis. Am. J. Transplant..

[200] Arkadopoulos N., Nastos C., Kalimeris K., Economou E., Theodoraki K., Kouskouni E., Pafiti A., Kostopanagiotou G., Smyrniotis V. (2010). Iron chelation for amelioration of liver ischemia-reperfusion injury. Hemoglobin.

[201] Shen H., Ma Y., Qiao Y., Zhang C., Chen J., Zhang R. (2024). Application of Deferoxamine in Tissue Regeneration Attributed to Promoted Angiogenesis. Molecules.

[202] Parker J.B., Griffin M.F., Downer M.A., Akras D., Berry C.E., Cotterell A.C., Gurtner G.C., Longaker M.T., Wan D.C. (2023). Chelating the valley of death: Deferoxamine’s path from bench to wound clinic. Front. Med..

[203] Chen Y., Li X., Wang S., Miao R., Zhong J. (2023). Targeting Iron Metabolism and Ferroptosis as Novel Therapeutic Approaches in Cardiovascular Diseases. Nutrients.

[204] Kontoghiorghes G., Marcus R.E., Huehns E.R. (1983). Desferrioxamine suppositories. Lancet.

[205] Gordon G.S., Ambruso D.R., Robinson W.A., Githens J.H. (1989). Intranasal administration of deferoxamine to iron overloaded patients. Am. J. Med. Sci..

[206] Farr A.C., Xiong M.P. (2021). Challenges and Opportunities of Deferoxamine Delivery for Treatment of Alzheimer’s Disease, Parkinson’s Disease, and Intracerebral Hemorrhage. Mol. Pharm..

[207] Agrawal M., Saraf S., Saraf S., Antimisiaris S.G., Chougule M.B., Shoyele S.A., Alexander A. (2018). Nose-to-brain drug delivery: An update on clinical challenges and progress towards approval of anti-Alzheimer drugs. J. Control. Release.

[208] Hanson L.R., Frey W.H. (2008). Intranasal delivery bypasses the blood-brain barrier to target therapeutic agents to the central nervous system and treat neurodegenerative disease. BMC Neurosci..

[209] Rao I.Y., Hanson L.R., Johnson J.C., Rosenbloom M.H., Frey W.H. (2022). Brain Glucose Hypometabolism and Iron Accumulation in Different Brain Regions in Alzheimer’s and Parkinson’s Diseases. Pharmaceuticals.

[210] McLachlan D.R.C., Dalton A.J., Kruck T.P., Bell M.Y., Smith W.L., Kalow W., Andrews D.F. (1991). Intramuscular desferrioxamine in patients with Alzheimer’s disease. Lancet.

[211] Donfrancesco A., Deb G., Dominici C., Angioni A., Caniglia M., De Sio L., Fidani P., Amici A., Helson L. (1992). Deferoxamine, cyclophosphamide, etoposide, carboplatin, and thiotepa (D-CECaT): A new cytoreductive chelation-chemotherapy regimen in patients with advanced neuroblastoma. Am. J. Clin. Oncol..

[212] Yamasaki T., Terai S., Sakaida I. (2011). Deferoxamine for advanced hepatocellular carcinoma. N. Engl. J. Med..

[213] Dreicer R., Kemp J.D., Stegink L.D., Cardillo T., Davis C.S., Forest P.K., See W.A. (1997). A phase II trial of deferoxamine in patients with hormone-refractory metastatic prostate cancer. Cancer Investig..

[214] Halliwell B., Gutteridge J.M., Blake D. (1985). Metal ions and oxygen radical reactions in human inflammatory joint disease. Philos. Trans. R. Soc. London. B Biol. Sci..

[215] Pambianchi E., Ferrara F., Pecorelli A., Benedusi M., Choudhary H., Therrien J.P., Valacchi G. (2021). Deferoxamine Treatment Improves Antioxidant Cosmeceutical Formulation Protection against Cutaneous Diesel Engine Exhaust Exposure. Antioxidants.

[216] Wu V.C., Wang R., Lai T.S., Wu K.D. (2006). Deferoxamine-related fatal nasal-orbital-cerebral mucormycosis. Kidney Int..

[217] Niihara Y., Ge J., Shalev O., Wu H., Tu A., Tanaka K.R. (2002). Desferrioxamine decreases NAD redox potential of intact red blood cells: Evidence for desferrioxamine as an inducer of oxidant stress in red blood cells. BMC Clin. Pharmacol..

[218] Jia H., Liu X., Cao Y., Niu H., Zhang L., Li R., Li F., Sun D., Shi M., Wa L. (2023). Deferoxamine ameliorates neurological dysfunction by inhibiting ferroptosis and neuroinflammation after traumatic brain injury. Brain Res..

[219] Wang H., Wu S., Li Q., Sun H., Wang Y. (2025). Targeting Ferroptosis: Acteoside as a Neuroprotective Agent in Salsolinol-Induced Parkinson’s Disease Models. Front. Biosci..

[220] Zhu D., Liang R., Liu Y., Li Z., Cheng L., Ren J., Guo Y., Wang M., Chai H., Niu Q. (2022). Deferoxamine ameliorated Al(mal)_3_-induced neuronal ferroptosis in adult rats by chelating brain iron to attenuate oxidative damage. Toxicol. Mech. Methods.

[221] Thorwald M.A., Godoy-Lugo J.A., Garcia G., Silva J., Kim M., Christensen A., Mack W.J., Head E., O’Day P.A., Benayoun B.A. (2025). Iron-associated lipid peroxidation in Alzheimer’s disease is increased in lipid rafts with decreased ferroptosis suppressors, tested by chelation in mice. Alzheimer’s Dement..

[222] Zhang Y., Du W., Kong T., Hua T., Ma H., Hu Y., Pan S., Ling B., Yang M., Cheng C. (2025). Targeted temperature management alleviates post-resuscitation myocardial dysfunction by inhibiting ferroptosis. Cell Death Discov..

[223] Kumfu S., Sripetchwandee J., Thonusin C., Sumneang N., Maneechote C., Arunsak B., Chunchai T., Oo T.T., Kongkaew A., Chattipakorn S.C. (2023). Ferroptosis inhibitor improves cardiac function more effectively than inhibitors of apoptosis and necroptosis through cardiac mitochondrial protection in rats with iron-overloaded cardiomyopathy. Toxicol. Appl. Pharmacol..

[224] Wang J., Wang Y., Liu Y., Cai X., Huang X., Fu W., Wang L., Qiu L., Li J., Sun L. (2022). Ferroptosis, a new target for treatment of renal injury and fibrosis in a 5/6 nephrectomy-induced CKD rat model. Cell Death Discov..

[225] Cao Y., Yang H., Huang Y., Lu J., Du H., Wang B. (2024). Mesenchymal stem cell-derived exosomal miR-26a induces ferroptosis, suppresses hepatic stellate cell activation, and ameliorates liver fibrosis by modulating SLC7A11. Open Med..

[226] Tang Q., Wang Y., Yan B., Zhang J., Wang T., Fang Y., Ye Z., Zhang N., Zhang N., Wu Z. (2024). Intracellular Magnetic Hyperthermia Sensitizes Sorafenib to Orthotopic Hepatocellular Carcinoma Via Amplified Ferroptosis. ACS Nano.

[227] Zhang S., Chang W., Wu H., Wang Y.H., Gong Y.W., Zhao Y.L., Liu S.H., Wang H.Z., Svatek R.S., Rodriguez R. (2020). Pan-cancer analysis of iron metabolic landscape across the Cancer Genome Atlas. J. Cell. Physiol..

[228] Kumada H., Itoh M., Tohda S. (2024). Effect of Ferroptosis Inducers and Inhibitors on Cell Proliferation in Acute Leukemia. Anticancer Res..

[229] Wang L., Zhang X., Xu M., Zheng G., Chen J., Li S., Cui J., Zhang S. (2023). Implication of ferroptosis in hepatic toxicity upon single or combined exposure to polystyrene microplastics and cadmium. Environ. Pollut..

[230] Wang Y., Wu J., Zhang M., OuYang H., Li M., Jia D., Wang R., Zhou W., Liu H., Hu Y. (2023). Cadmium exposure during puberty damages testicular development and spermatogenesis via ferroptosis caused by intracellular iron overload and oxidative stress in mice. Environ. Pollut..

[231] Pan L., Yu Z., Xiang W.X., Sun S.R., Li J.X., Deng Y.K., Wang M.C., Zhong J.X., Huang K., Gao P.S. (2025). Cigarette smoke-induced epithelial cell ferroptosis promotes neutrophilic inflammation in patients with nasal polyps. J. Allergy Clin. Immunol..

[232] Walter P.B., Macklin E.A., Porter J., Evans P., Kwiatkowski J.L., Neufeld E.J., Coates T., Giardina P.J., Vichinsky E., Olivieri N. (2008). Inflammation and oxidant-stress in beta-thalassemia patients treated with iron chelators deferasirox (ICL670) or deferoxamine: An ancillary study of the Novartis CICL670A0107 trial. Haematologica.

[233] Murillo Ortiz B.O., Ramírez Emiliano J., Romero Vázquez M.J., Amador Medina L.F., Martínez Garza S., Ramos Rodríguez E.M. (2025). Impact of iron chelation with deferasirox on telomere length and oxidative stress in hemodialysis patients: A randomized study. Nefrologia.

[234] Ghoti H., Fibach E., Merkel D., Perez-Avraham G., Grisariu S., Rachmilewitz E.A. (2010). Changes in parameters of oxidative stress and free iron biomarkers during treatment with deferasirox in iron-overloaded patients with myelodysplastic syndromes. Haematologica.

[235] Neaimy K., Alsarraf O., Alkhyatt M. (2024). Comparative Study of Oxidative Stress in Patients with Β-Thalassemia Major on Deferasirox Versus Deferoxamine Therapy. Georgian Med. News.

[236] Belini Junior E., da Silva D.G., Torres Lde S., de Almeida E.A., Cancado R.D., Chiattone C., Bonini-Domingos C.R. (2012). Oxidative stress and antioxidant capacity in sickle cell anaemia patients receiving different treatments and medications for different periods of time. Ann. Hematol..

[237] Menaker N., Halligan K., Shur N., Paige J., Hickling M., Nepo A., Weintraub L. (2017). Acute Liver Failure During Deferasirox Chelation: A Toxicity Worth Considering. J. Pediatr. Hematol. Oncol..

[238] Caranfa J., Carrera W., Marmalidou A., Desai S., Baumal C. (2024). Multimodal imaging in deferasirox-mediated retinopathy. Eur. J. Ophthalmol..

[239] Scoglio M., Cappellini M.D., D’Angelo E., Bianchetti M.G., Lava S.A.G., Agostoni C., Milani G.P. (2021). Kidney Tubular Damage Secondary to Deferasirox: Systematic Literature Review. Children.

[240] Badeli H., Baghersalimi A., Eslami S., Saadat F., Rad A.H., Basavand R., Papkiadeh S.R., Darbandi B., Kooti W., Peluso I. (2019). Early Kidney Damage Markers after Deferasirox Treatment in Patients with Thalassemia Major: A Case-Control Study. Oxidative Med. Cell. Longev..

[241] García-Fariña B., Rink L., Santarini V., Westkemper M., Dohna-Schwake C., Möhlendick B. (2024). Case report: Acute liver failure during deferasirox therapy and the potential role of pharmacogenetics. Front. Pharmacol..

[242] Delgado Y., Torres-Sanchez A., Perez D., Torres G., Estrada S., Ortiz Alvelo N., Vega J., Santos L., Torres A., Madera B.A. (2024). Deferasirox’s Anti-Chemoresistance and Anti-Metastatic Effect on Non-Small Cell Lung Carcinoma. Biomedicines.

[243] Mo M., Pan L., Deng L., Liang M., Xia N., Liang Y. (2024). Iron Overload Induces Hepatic Ferroptosis and Insulin Resistance by Inhibiting the Jak2/stat3/slc7a11 Signaling Pathway. Cell Biochem. Biophys..

[244] Chen Y., Li Y., Wu M., Li Z. (2024). Electroacupuncture improves cognitive function in APP/PS1 mice by inhibiting oxidative stress related hippocampal neuronal ferroptosis. Brain Res..

[245] Hsu W.Y., Wang L.T., Lin P.C., Liao Y.M., Hsu S.H., Chiou S.S. (2024). Deferasirox Causes Leukaemia Cell Death through Nrf2-Induced Ferroptosis. Antioxidants.

[246] Ishimaru K., Ikeda M., Miyamoto H.D., Furusawa S., Abe K., Watanabe M., Kanamura T., Fujita S., Nishimura R., Toyohara T. (2024). Deferasirox Targeting Ferroptosis Synergistically Ameliorates Myocardial Ischemia Reperfusion Injury in Conjunction with Cyclosporine A. J. Am. Heart Assoc..

[247] Thapa K., Singh T.G., Kaur A. (2022). Targeting ferroptosis in ischemia/reperfusion renal injury. Naunyn Schmiedeberg’s Arch. Pharmacol..

[248] Wu Y., Ran L., Yang Y., Gao X., Peng M., Liu S., Sun L., Wan J., Wang Y., Yang K. (2023). Deferasirox alleviates DSS-induced ulcerative colitis in mice by inhibiting ferroptosis and improving intestinal microbiota. Life Sci..

[249] Timoshnikov V.A., Kichigina L.A., Selyutina O.Y., Polyakov N.E., Kontoghiorghes G.J. (2021). Antioxidant Activity of Deferasirox and Its Metal Complexes in Model Systems of Oxidative Damage: Comparison with Deferiprone. Molecules.

[250] Pippard M.J., Jackson M.J., Hoffman K., Petrou M., Modell C.B. (1986). Iron chelation using subcutaneous infusions of diethylene triamine penta-acetic acid (DTPA). Scand. J. Haematol..

[251] Born T., Kontoghiorghe C.N., Spyrou A., Kolnagou A., Kontoghiorghes G.J. (2013). EDTA chelation reappraisal following new clinical trials and regular use in millions of patients: Review of preliminary findings and risk/benefit assessment. Toxicol. Mech. Methods.

[252] Fan W., Guo M. (2025). Research progress on nanotoxicity and detoxification of cobalt in metal-based implants. Ann Med..

[253] Dumala N., Chintala S., Mangalampalli B., Venkata R.P. (2025). Elucidation of 28 day repeated oral dose induced genotoxicity potential of nickel (II) oxide nanoparticles in wistar albino rats. Regul Toxicol Pharmacol..

[254] Mantle D., Golomb B.A. (2025). Coenzyme Q10 and Xenobiotic Metabolism: An Overview. Int J Mol Sci..

[255] Arruebarrena M.A., Hawe C.T., Lee Y.M., Branco R.C. (2023). Mechanisms of Cadmium Neurotoxicity. Int. J. Mol. Sci..

[256] Wax P.M. (2013). Current use of chelation in American health care. J. Med. Toxicol..

[257] Lamas G.A., Goertz C., Boineau R., Mark D.B., Rozema T., Nahin R.L., Lindblad L., Lewis E.F., Drisko J., Lee K.L. (2013). Effect of disodium EDTAchelation regimen on cardiovascular events in patients with previous myocardial infarction: The TACTrandomized trial. JAMA.

[258] Ravalli F., Vela Parada X., Ujueta F., Pinotti R., Anstrom K.J., Lamas G.A., Navas-Acien A. (2022). Chelation Therapy in Patients with Cardiovascular Disease: A Systematic Review. J. Am. Heart Assoc..

[259] Ujueta F., Lamas G.A., Anstrom K.J., Navas-Acien A., Boineau R., Rosenberg Y., Stylianou M., Jones T.L.Z., Joubert B.R., Yu Q. (2025). Multivitamins After Myocardial Infarction in Patients with Diabetes: A Randomized Clinical Trial. JAMA Intern. Med..

[260] Vezzoli A., Mrakic-Sposta S., Dellanoce C., Montorsi M., Vietti D., Ferrero M.E. (2023). Chelation Therapy Associated with Antioxidant Supplementation Can Decrease Oxidative Stress and Inflammation in Multiple Sclerosis: Preliminary Results. Antioxidants.

[261] Fulgenzi A., Vietti D., Ferrero M.E. (2020). EDTA Chelation Therapy in the Treatment of Neurodegenerative Diseases: An Update. Biomedicines.

[262] Baxter A.J., Krenzelok E.P. (2008). Pediatric fatality secondary to EDTA chelation. Clin. Toxicol..

[263] Discalzi G., Pira E., Herrero Hernandez E., Valentini C., Turbiglio M., Meliga F. (2000). Occupational Mn parkinsonism: Magneticresonance imaging and clinical patterns following CaNa2-EDTA chelation. Neurotoxicology.

[264] Brown M.J., Willis T., Omalu B., Leiker R. (2006). Deaths resulting from hypocalcemia after administration of edetate disodium: 2003-2005. Pediatrics.

[265] Morgan B.W., Kori S., Thomas J.D. (2002). Adverse effects in 5 patients receiving EDTA at an outpatient chelation clinic. Vet. Hum. Toxicol..

[266] Hininger I., Waters R., Osman M., Garrel C., Fernholz K., Roussel A.M., Anderson R.A. (2005). Acute prooxidant effects of vitamin C in EDTA chelation therapy and long-term antioxidant benefits of therapy. Free Radic. Biol. Med..

[267] Roussel A.M., Hininger-Favier I., Waters R.S., Osman M., Fernholz K., Anderson R.A. (2009). EDTA chelation therapy, withoutadded vitamin C, decreases oxidative DNA damage and lipid peroxidation. Altern. Med. Rev..

[268] Samuni Y., Goldstein S., Dean O.M., Berk M. (2013). The chemistry and biological activities of N-acetylcysteine. Biochim. Biophys. Acta.

[269] Olsson B., Johansson M., Gabrielsson J., Bolme P. (1988). Pharmacokinetics and bioavailability of reduced and oxidized N-acetylcysteine. Eur. J. Clin. Pharmacol..

[270] Bailey G.P., Wood D.M., Archer J.R., Rab E., Flanagan R.J., Dargan P.I. (2017). An assessment of the variation in the concentration of acetylcysteine in infusions for the treatment of paracetamol overdose. Br. J. Clin. Pharmacol..

[271] Clark R.S.B., Empey P.E., Bayır H., Rosario B.L., Poloyac S.M., Kochanek P.M., Nolin T.D., Au A.K., Horvat C.M., Wisniewski S.R. (2017). Phase I randomized clinical trial of N-acetylcysteine in combination with an adjuvant probenecid for treatment of severe traumatic brain injury in children. PLoS ONE.

[272] Conus P., Seidman L.J., Fournier M., Xin L., Cleusix M., Baumann P.S., Ferrari C., Cousins A., Alameda L., Gholam-Rezaee M. (2018). N-acetylcysteine in a Double-Blind Randomized Placebo-Controlled Trial: Toward Biomarker-Guided Treatment in Early Psychosis. Schizophr. Bull..

[273] Zhang Y., Ding S., Li C. (2017). Effects of N-acetylcysteine treatment in acute respiratory distress syndrome: A meta-analysis. Exp. Ther. Med..

[274] Tenório M.B., Ferreira R.C., Moura F.A., Bueno N., Goulart M., Oliveira A. (2018). Oral antioxidant therapy for prevention and treatment of preeclampsia: Meta-analysis of randomized controlled trials. Nutr. Metab. Cardiovasc. Dis..

[275] Modarresi A., Ziaie S., Salamzadeh J., Sahraei Z., Nafar M., Panahi Y., Parvin M., Einollahi B. (2017). Study of The Effects of N-Acetylcysteine on Oxidative Stress Status of Patients on Maintenance-Hemodialysis Undergoing Cadaveric Kidney Transplantation. Iran. J. Pharm. Res..

[276] Wallis R.S., Sabi I., Lalashowi J., Bakuli A., Mapamba D., Olomi W., Siyame E., Ngaraguza B., Chimbe O., Charalambous S. (2024). Adjunctive N-Acetylcysteine and Lung Function in Pulmonary Tuberculosis. NEJM Evid..

[277] Komakula S., Bhatia R., Sahib A., Upadhyay A., S L.J., Garg A., Y V.V., Pandit A.K., Vibha D., Singh M.B. (2024). Safety and efficacy of N-acetylcysteine (NAC) as an adjunct to standard treatment in patients with acute ischemic stroke: A randomized controlled pilot trial (NACTLYS). Sci. Rep..

[278] Zhang Q., Liu Z., Wang T., Yu M., Li X. (2024). Efficacy and acceptability of adjunctive n-acetylcysteine for psychotic disorders: Systematic review and meta-analysis. Hum. Psychopharmacol..

[279] Babu Balagopal P., Kohli R., Uppal V., Averill L., Shah C., McGoogan K., Di Guglielmo M., Goran M., Hossain M.J. (2024). Effect of N-acetyl cysteine in children with metabolic dysfunction-associated steatotic liver disease-A pilot study. J. Pediatr. Gastroenterol. Nutr..

[280] Soleimani A., Habibi M.R., Hasanzadeh Kiabi F., Alipour A., Habibi V., Azizi S., Zeydi A.E., Sohrabi F.B. (2018). The effect of intravenous N-acetylcysteine on prevention of atrial fibrillation after coronary artery bypass graft surgery: A double-blind, randomised, placebo-controlled trial. Kardiol. Pol..

[281] Sharafkhah M., Abdolrazaghnejad A., Zarinfar N., Mohammadbeigi A., Massoudifar A., Abaszadeh S. (2018). Safety and efficacy of N-acetyl-cysteine for prophylaxis of ventilator-associated pneumonia: A randomized, double blind, placebo-controlled clinical trial. Med. Gas Res..

[282] Mantzarlis K., Tsolaki V., Zakynthinos E. (2017). Role of Oxidative Stress and Mitochondrial Dysfunction in Sepsis and Potential Therapies. Oxidative Med. Cell. Longev..

[283] Bavarsad Shahripour R., Harrigan M.R., Alexandrov A.V. (2014). N-acetylcysteine (NAC) in neurological disorders: Mechanisms of action and therapeutic opportunities. Brain Behav..

[284] Wong G., Wu S.Y., Chen W.M., Hsu P.J., Chou T.C., Chiang M.F., Wu M.S., Lee M.C., Soong R.S. (2024). Effects of N-acetylcysteine on hepatocellular carcinoma in chronic hepatitis C. Am. J. Cancer Res..

[285] van Zandwijk N., Dalesio O., Pastorino U., de Vries N., van Tinteren H. (2000). EUROSCAN, a randomized trial of vitamin A and N-acetylcysteine in patients with head and neck cancer or lung cancer. For the EUropean Organization for Research and Treatment of Cancer Head and Neck and Lung Cancer Cooperative Groups. J. Natl. Cancer Inst..

[286] Cassidy P.B., Liu T., Florell S.R., Honeggar M., Leachman S.A., Boucher K.M., Grossman D. (2017). A Phase II Randomized Placebo-Controlled Trial of Oral N-acetylcysteine for Protection of Melanocytic Nevi against UV-Induced Oxidative Stress In Vivo. Cancer Prev. Res..

[287] Posadzki P., Lee M.S., Onakpoya I., Lee H.W., Ko B.S., Ernst E. (2013). Dietary supplements and prostate cancer: A systematic review of double-blind, placebo-controlled randomised clinical trials. Maturitas.

[288] Monti D., Sotgia F., Whitaker-Menezes D., Tuluc M., Birbe R., Berger A., Lazar M., Cotzia P., Draganova-Tacheva R., Lin Z. (2017). Pilot study demonstrating metabolic and anti-proliferative effects of in vivo anti-oxidant supplementation with N-Acetylcysteine in Breast Cancer. Semin. Oncol..

[289] Tsang R.Y., Al-Fayea T., Au H.J. (2009). Cisplatin overdose: Toxicities and management. Drug Saf..

[290] Cloos J., Bongers V., Lubsen H., Tobi H., Braakhuis B.J., Snow G.B. (1996). Lack of effect of daily N-acetylcysteine supplementation on mutagen sensitivity. Cancer Epidemiol. Biomarkers Prev..

[291] Block K.I., Koch A.C., Mead M.N., Tothy P.K., Newman R.A., Gyllenhaal C. (2008). Impact of antioxidant supplementation on chemotherapeutic toxicity: A systematic review of the evidence from randomized controlled trials. Int. J. Cancer.

[292] Orgel E., Knight K.R., Chi Y.Y., Malvar J., Rushing T., Mena V., Eisenberg L.S., Rassekh S.R., Ross C.J.D., Scott E.N. (2023). Intravenous N-Acetylcysteine to Prevent Cisplatin-Induced Hearing Loss in Children: A Nonrandomized Controlled Phase I Trial. Clin. Cancer Res..

[293] Zavala-Valencia A.C., Velasco-Hidalgo L., Martínez-Avalos A., Castillejos-López M., Torres-Espíndola L.M. (2024). Effect of N-Acetylcysteine on Cisplatin Toxicity: A Review of the Literature. Biologics.

[294] Sins J.W.R., Fijnvandraat K., Rijneveld A.W., Boom M.B., Kerkhoffs J.H., van Meurs A.H., de Groot M.R., Heijboer H., Dresse M.F., Lê P.Q. (2018). Effect of N-acetylcysteine on pain in daily life in patients with sickle cell disease: A randomised clinical trial. Br. J. Haematol..

[295] Ozdemir Z.C., Koc A., Aycicek A., Kocyigit A. (2014). N-Acetylcysteine supplementation reduces oxidative stress and DNA damage in children with β-thalassemia. Hemoglobin.

[296] Amen F., Machin A., Touriño C., Rodríguez I., Denicola A., Thomson L. (2017). N-acetylcysteine improves the quality of red blood cells stored for transfusion. Arch. Biochem. Biophys..

[297] Ghazaiean M., Aliasgharian A., Karami H., Ghasemi M.M., Darvishi-Khezri H. (2024). Antioxidative effects of N-acetylcysteine in patients with β-thalassemia: A quick review on clinical trials. Health Sci. Rep..

[298] Bolarinwa A.B., Oduwole O., Okebe J., Ogbenna A.A., Otokiti O.E., Olatinwo A.T. (2024). Antioxidant supplementation for sickle cell disease. Cochrane Database Syst. Rev..

[299] Zheng J., Zhang W., Ito J., Henkelmann B., Xu C., Mishima E., Conrad M. (2025). N-acetyl-l-cysteine averts ferroptosis by fostering glutathione peroxidase 4. Cell Chem. Biol..

[300] Hu M., Zhang Y., Ma S., Li J., Wang X., Liang M., Sferruzzi-Perri A.N., Wu X., Ma H., Brännström M. (2021). Suppression of uterine and placental ferroptosis by N-acetylcysteine in a rat model of polycystic ovary syndrome. Mol. Hum. Reprod..

[301] Wu Z., Zhu Y., Liu W., Balasubramanian B., Xu X., Yao J., Lei X. (2024). Ferroptosis in Liver Disease: Natural Active Compounds and Therapeutic Implications. Antioxidants.

[302] Karuppagounder S.S., Alin L., Chen Y., Brand D., Bourassa M.W., Dietrich K., Wilkinson C.M., Nadeau C.A., Kumar A., Perry S. (2018). N-acetylcysteine targets 5 lipoxygenase-derived, toxic lipids and can synergize with prostaglandin E_2_ to inhibit ferroptosis and improve outcomes following hemorrhagic stroke in mice. Ann. Neurol..

[303] Gao C., Wang L., Fu K., Cheng S., Wang S., Feng Z., Yu S., Yang Z. (2025). N-Acetylcysteine Alleviates Necrotizing Enterocolitis by Depressing SESN2 Expression to Inhibit Ferroptosis in Intestinal Epithelial Cells. Inflammation.

[304] Zhou D., Yang Y., Chen J., Zhou J., He J., Liu D., Zhang A., Yuan B., Jiang Y., Xia W. (2024). N-acetylcysteine Protects Against Myocardial Ischemia-Reperfusion Injury Through Anti-ferroptosis in Type 1 Diabetic Mice. Cardiovasc. Toxicol..

[305] Kalyanaraman B. (2022). NAC, NAC, Knockin’ on Heaven’s door: Interpreting the mechanism of action of N-acetylcysteine in tumor and immune cells. Redox Biol..

[306] Ding Z., Li Z., Sun K., Liu Y., Fang Z., Sun S., Li C., Wang Z. (2025). Mitochondrial Regulation of Ferroptosis in Cancer Cells. Int. J. Biol. Sci..

[307] Bebber C.M., Müller F., Prieto Clemente L., Weber J., von Karstedt S. (2020). Ferroptosis in Cancer Cell Biology. Cancers.

[308] Jiang Y., Glandorff C., Sun M. (2024). GSH and Ferroptosis: Side-by-Side Partners in the Fight against Tumors. Antioxidants.

[309] Kruck T.P., Kalow W., McLachlan D.R. (1985). Determination of desferoxamine and a major metabolite by high-performance liquid chromatography. Application to the treatment of aluminium-related disorders. J. Chromatogr..

[310] Singh S., Hider R.C., Porter J.B. (1990). Separation and identification of desferrioxamine and its iron chelating metabolites by high-performance liquid chromatography and fast atom bombardment mass spectrometry: Choice of complexing agent and application to biological fluids. Anal. Biochem..

[311] Kontoghiorghes G.J., Kolnagou A., Kontoghiorghe C.N., Mourouzidis L., Timoshnikov V.A., Polyakov N.E. (2020). Trying to Solve the Puzzle of the Interaction of Ascorbic Acid and Iron: Redox, Chelation and Therapeutic Implications. Medicines.

[312] Levy G. (1980). Clinical pharmacokinetics of salicylates: A re-assessment. Br. J. Clin. Pharmacol..

[313] Navarro S.L., Saracino M.R., Makar K.W., Thomas S.S., Li L., Zheng Y., Levy L., Schwarz Y., Bigler J., Potter J.D. (2011). Determinants of aspirin metabolism in healthy men and women: Effects of dietary inducers of UDP-glucuronosyltransferases. Lifestyle Genom..

[314] Kontoghiorghes G.J. (2024). The Puzzle of Aspirin and Iron Deficiency: The Vital Missing Link of the Iron-Chelating Metabolites. Int. J. Mol. Sci..

[315] Malisza K.L., Hasinoff B.B. (1995). Doxorubicin reduces the iron(III) complexes of the hydrolysis products of the antioxidant cardioprotective agent dexrazoxane (ICRF-187) and produces hydroxyl radicals. Arch. Biochem. Biophys..

[316] Hasinoff B.B. (1998). Chemistry of dexrazoxane and analogues. Semin. Oncol..

[317] Jirkovský E., Jirkovská A., Bureš J., Chládek J., Lenčová O., Stariat J., Pokorná Z., Karabanovich G., Roh J., Brázdová P. (2018). Pharmacokinetics of the Cardioprotective Drug Dexrazoxane and Its Active Metabolite ADR-925 with Focus on Cardiomyocytes and the Heart. J. Pharmacol. Exp. Ther..

[318] Tolani D., Wilcox J., Shyam S., Bansal N. (2023). Cardio-oncology for Pediatric and Adolescent/Young Adult Patients. Curr. Treat. Options Oncol..

[319] Spalato Ceruso M., Napolitano A., Silletta M., Mazzocca A., Valeri S., Improta L., Santini D., Tonini G., Badalamenti G., Vincenzi B. (2019). Use of Cardioprotective Dexrazoxane Is Associated with Increased Myelotoxicity in Anthracycline-Treated Soft-Tissue Sarcoma Patients. Chemotherapy.

[320] Jones R.L. (2008). Utility of dexrazoxane for the reduction of anthracycline-induced cardiotoxicity. Expert Rev. Cardiovasc. Ther..

[321] Barnabé N., Zastre J.A., Venkataram S., Hasinoff B.B. (2002). Deferiprone protects against doxorubicin-induced myocyte cytotoxicity. Free Radic. Biol. Med..

[322] Kontoghiorghes G.J. (2025). New Insights into Aspirin’s Anticancer Activity: The Predominant Role of Its Iron-Chelating Antioxidant Metabolites. Antioxidants.

[323] Guirguis-Blake J.M., Evans C.V., Perdue L.A., Bean S.I., Senger C.A. (2022). Aspirin Use to Prevent Cardiovascular Disease and Colorectal Cancer: Updated Evidence Report and Systematic Review for the US Preventive Services Task Force. JAMA.

[324] Nafisi S., Støer N.C., Veierød M.B., Randel K.R., Hoff G., Löfling L., Bosetti C., Botteri E. (2024). Low-Dose Aspirin and Prevention of Colorectal Cancer: Evidence From a Nationwide Registry-Based Cohort in Norway. Am. J. Gastroenterol..

[325] Sikavi D.R., Wang K., Ma W., Drew D.A., Ogino S., Giovannucci E.L., Cao Y., Song M., Nguyen L.H., Chan A.T. (2024). Aspirin Use and Incidence of Colorectal Cancer According to Lifestyle Risk. JAMA Oncol..

[326] Lloyd K.E., Hall L.H., Ziegler L., Foy R., Green S.M.C., MacKenzie M., Taylor D.G., Smith S.G., Aspirin for Cancer Prevention AsCaP Steering Committee (2023). Acceptability of aspirin for cancer preventive therapy: A survey and qualitative study exploring the views of the UK general population. BMJ Open.

[327] Lin J.R., Han D.D., Wei W., Zeng Q., Rong Z.X., Bai X., Zhang Y.P., Wang J., Cai X.T., Rao X.G. (2024). Regular use of aspirin and statins reduces the risk of cancer in individuals with systemic inflammatory diseases. Cancer Res..

[328] Zheng G., Faber M.T., Wang J., Baandrup L., Hertzum-Larsen R., Sundström K., Kjær S.K. (2024). Low-dose aspirin use and risk of ovarian cancer: A combined analysis from two nationwide studies in Denmark and Sweden. Br. J. Cancer.

[329] Sassano M., Taborelli M., Boccia S., Cadoni G., La Vecchia C., Garavello W., Lazarus P., Lee Y.A., Hashibe M., Boffetta P. (2024). Aspirin intake and head and neck cancer: A pooled analysis within the INHANCE consortium. Head Neck.

[330] Yang J., Yamashita-Kanemaru Y., Morris B.I., Contursi A., Trajkovski D., Xu J., Patrascan I., Benson J., Evans A.C., Conti A.G. (2025). Aspirin prevents metastasis by limiting platelet TXA_2_ suppression of T cell immunity. Nature.

[331] Chen H., Qi Q., Wu N., Wang Y., Feng Q., Jin R., Jiang L. (2022). Aspirin promotes RSL3-induced ferroptosis by suppressing mTOR/SREBP-1/SCD1-mediated lipogenesis in PIK3CA-mutant colorectal cancer. Redox Biol..

[332] Wu Z., Li D., Tian D., Liu X., Wu Z. (2022). Aspirin mediates protection from diabetic kidney disease by inducing ferroptosis inhibition. PLoS ONE.

[333] Wang Y.F., Feng J.Y., Zhao L.N., Zhao M., Wei X.F., Geng Y., Yuan H.F., Hou C.Y., Zhang H.H., Wang G.W. (2023). Aspirin triggers ferroptosis in hepatocellular carcinoma cells through restricting NF-κB p65-activated SLC7A11 transcription. Acta Pharmacol. Sin..

[334] Zhao L., Zhou X., Xie F., Zhang L., Yan H., Huang J., Zhang C., Zhou F., Chen J., Zhang L. (2022). Ferroptosis in cancer and cancer immunotherapy. Cancer Commun..

[335] Zhang Y., Xie J. (2024). Targeting ferroptosis regulators by natural products in colorectal cancer. Front. Pharmacol..

[336] Kontoghiorghes G.J. (2023). Deferiprone and Iron-Maltol: Forty Years since Their Discovery and Insights into Their Drug Design, Development, Clinical Use and Future Prospects. Int. J. Mol. Sci..

[337] Kontoghiorghes G.J., Kolnagou A., Demetriou T., Neocleous M., Kontoghiorghe C.N. (2021). New Era in the Treatment of Iron Deficiency Anaemia Using Trimaltol Iron and Other Lipophilic Iron Chelator Complexes: Historical Perspectives of Discovery and Future Applications. Int. J. Mol. Sci..

[338] Jung M., Mertens C., Tomat E., Brüne B. (2019). Iron as a Central Player and Promising Target in Cancer Progression. Int. J. Mol. Sci..

[339] Kontoghiorghes G.J., Agarwal M.B., Tondury P., Kersten M.J., Jaeger M., Vreugdenhil G., Vania A., Rahman Y.E. (1993). Future of oral iron chelator deferiprone. (L1) ICOC committee. Lancet.

[340] Kolnagou A., Kontoghiorghes G.J. (2006). Effective combination therapy of deferiprone and deferoxamine for the rapid clearance of excess cardiac IRON and the prevention of heart disease in thalassemia. The Protocol of the International Committee on Oral Chelators. Hemoglobin.

